# CLEC-1 promotes early IFN response in cDC1s and favors immune dysregulation during sepsis in mice

**DOI:** 10.1016/j.isci.2026.117239

**Published:** 2026-08-24

**Authors:** Thomas Teja Ogor, Esther Porée, Javier Saenz, Marion Davieau, Victor Gourain, Camille Ligeron, Florian Pierre Martin, Cynthia Fourgeux, Claire Chédeville, Julie Mignon, Martin Braud, Aurélie Moreau, Régis Josien, Aurore Morello, Etienne Foucher, Nicolas Poirier, Jeremie Poschmann, Antoine Roquilly, Cédric Jacqueline, Elise Chiffoleau

**Affiliations:** 1 Nantes Université, INSERM, Center for Research in Transplantation and Translational Immunology, UMR 1064, 44000 Nantes, France; 2 CHU Nantes, INSERM, Nantes Université, Anesthesie Reanimation, CIC 1413, 44000 Nantes, France; 3 Ose Immunotherapeutics, Nantes, France; 4 Nantes Université, CHU Nantes, Laboratoire d’Immunologie Biologique, Nantes, France

**Keywords:** inflammation, chemokines, neutrophils, monocytes/macrophages, immunotherapy, rodent

## Abstract

Sepsis is a life-threatening organ dysfunction caused by a dysregulated host immune response, characterized by both hyperinflammation and immunosuppression. Here, we show that the absence of the C-type lectin receptor CLEC-1—expressed particularly by lung cDC1s—improves mouse recovery following *E. coli* infection. Mechanistically, phenotypic and transcriptomic analyses revealed that *Clec1a* loss is characterized by an attenuated type I interferon response in lung cDC1s as well as reduced CXCL10 expression and monocyte infiltration during the acute phase of sepsis. Additionally, *Clec1a* deficiency is associated with an enhanced antigen presentation profile of lung myeloid cells and lower accumulation of regulatory T cells upon secondary infection, suggesting reduced immunoparalysis. Importantly, we demonstrate that blocking CLEC-1 using an anti-human CLEC-1 monoclonal antibody mitigates hyperinflammation in CLEC-1 humanized mice. Together, these findings uncover a role for CLEC-1 in the dysregulation of the inflammatory response during sepsis and suggest its potential as an immunotherapeutic target.

## Highlights

• Absence of *Clec1a* improves mouse recovery during sepsis
• Absence of *Clec1a* reduced CXCL10 and lung monocyte infiltration during sepsis
• Absence of *Clec1a* limits the early IFN response in lung cDC1s during sepsis

## Graphical abstract

**Figure:**
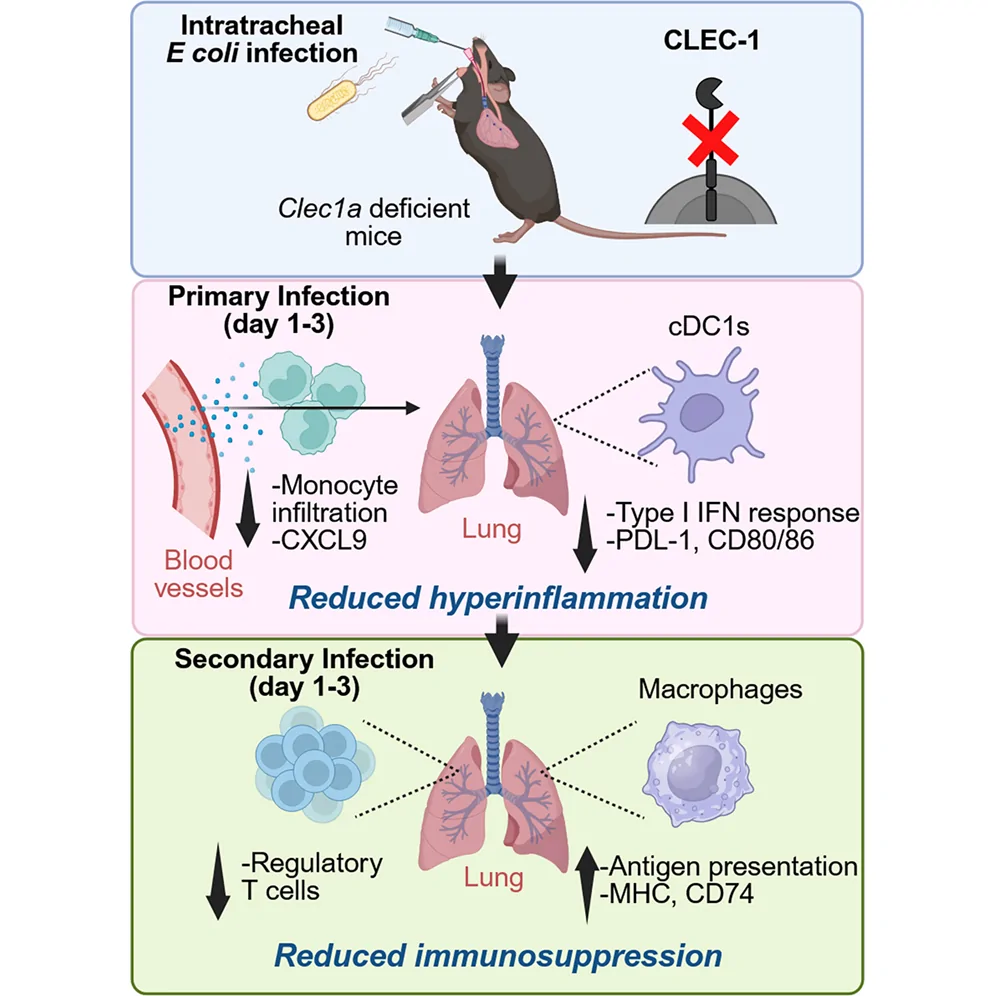

## Teaser

Biological sciences; Immunology; Omics

## Introduction

Sepsis is a multiorgan dysfunction that affects nearly 50 million people globally per year. An estimated 11 million people die from the disease, accounting for almost 20% of all deaths worldwide.^1^ Sepsis is characterized by an imbalanced host-to-infection immune response, wherein pathogens have evaded protective defense mechanisms and continue to multiply, resulting in persistent stimulation of host cells and injury, and a failure to return to homeostasis.^2^ In recent decades, significant advances have been made in the comprehension of the pathophysiology of sepsis,^2^^,^^3^ but treatment is still limited to pathogen control and supportive care. To date, few proven therapies address the intrinsic mechanisms underlying this life-threatening condition. Sepsis is characterized by a dysregulated host response to infection, with a strong host pro-inflammatory response combined with tissue damage, endothelial cell dysfunction, and organ failure. Concomitantly from the very onset of sepsis, immunosuppressive mechanisms are rapidly developed by innate and adaptative immune cells to limit inflammation, protect the host, and restore tissue homeostasis.^4^ In this unbalanced response, many of the immune mechanisms initially activated to provide protection become detrimental, both related to excessive inflammation and immunosuppression. Furthermore, if this dysregulation persists, it can lead to the development of severe immune defects in the hosts, a phenomenon known as sepsis-induced immunosuppression or immunoparalysis, which increases the susceptibility to secondary infections and mortality.^3^ Mechanisms involved in sepsis-induced immunosuppression have only started to be elucidated and relate to different cell types and features. Sepsis-induced immunosuppression is characterized by the abnormal death of immune effector cells, the release of anti-inflammatory cytokines, the hyperproliferation of immune suppressor cells, and the expression of immune checkpoint molecules.^5^ Moreover, homeostasis of myeloid cells from the lung is severely disrupted during sepsis. Monocytes/macrophages notably acquire epigenetic marks during lung infection, leading to their exhausted phenotype and impaired phagocytosis.^6^ In addition, dendritic cells (DCs) rapidly lose their capacity to secrete interleukin (IL) 12 and to present antigens, thereby limiting effector T cell priming while promoting regulatory T cell (Tregs) expansion and immunosuppression.^7^^,^^8^^,^^9^^,^^10^ It is therefore of interest to develop immunotherapies aimed at restoring myeloid cell function and pulmonary homeostasis during sepsis.

Given their ability to regulate myeloid cell activation, a growing body of evidence suggests that the innate C-type lectin receptors (CLRs) represent attractive therapeutic targets to modulate immune response.^11^^,^^12^^,^^13^ CLRs recognize not only pathogen-associated molecular patterns (PAMPs) for host defense, but also modified self-antigens such as damage-associated molecular patterns (DAMPs) released by injured cells. These receptors were shown to regulate numerous properties of myeloid cells such as antigen presentation, secretion of cytokines and chemokines, as well as type I interferon (IFN) response. They can act as activating or inhibitory receptors and are able to interfere with the signaling of other pattern recognition receptors (PRRs) to finely polarize subsequent T cell responses. As such, CLRs can dictate pathogen response and shape T cell immunity, modulating the balance between infection-driven inflammation and pathogen control.

We previously characterized the CLR CLEC-1 as particularly abundant in the lungs and as expressed by the cDC1 subset of myeloid cells and by endothelial cells.^14^^,^^15^^,^^16^ We showed that its expression in DCs is decreased by pro-inflammatory stimuli but enhanced by the immunosuppressive cytokine TGFβ. CLEC-1 belongs to the “Dectin” cluster of genes encoding MINCLE, DECTIN-1 and LOX-1. Although identified long ago, CLEC-1 remains the least characterized of this cluster, as functional work was restricted due to the lack of known ligands. However, CLEC-1 might be an important protein given its high evolutionary conservation between species, with 70% protein sequence similarity between human and mouse. CLEC-1 does not contain a classical immunoreceptor tyrosine-based activation (ITAM) or inhibition (ITIM) motif in the cytoplasmic tail but rather a tyrosine residue in a non-characterized signaling sequence [YSST], which could potentially be used for downstream signaling.^17^ We recently demonstrated that CLEC-1 is a receptor for necrotic cells and prevents collateral damage following tissue injury by tempering the early infiltration by neutrophils.^14^^,^^18^ Furthermore, we showed that CLEC-1 restricts antitumor immunity by limiting the cross-presentation of dead cell-associated antigens by cDC1s and by promoting the accumulation of immunosuppressive myeloid cells in tumors.^14^ However, its role in non-sterile inflammation remained to be explored. As DC functions are severely impaired during sepsis,^7^ we investigated the role of CLEC-1 in the dysregulation of the immune response occurring during this condition. For that, we used a well-established mouse model of sepsis induced by infection with *Escherichia coli (E. coli)*,^6^^,^^9^ and analyzed the transcriptomic and phenotypic changes over time during primary and secondary infections in the absence of CLEC-1 signaling. Unexpectedly, we found that the absence of CLEC-1 during sepsis improves mouse recovery and limits the early type I IFN response in pulmonary cDC1s and monocyte infiltration, suggesting that CLEC-1 could represent a target for immunotherapy.

## Results

### Absence of CLEC-1 improves mouse recovery and reduces CXCL10 and monocyte infiltration during sepsis

**Figure 1:**
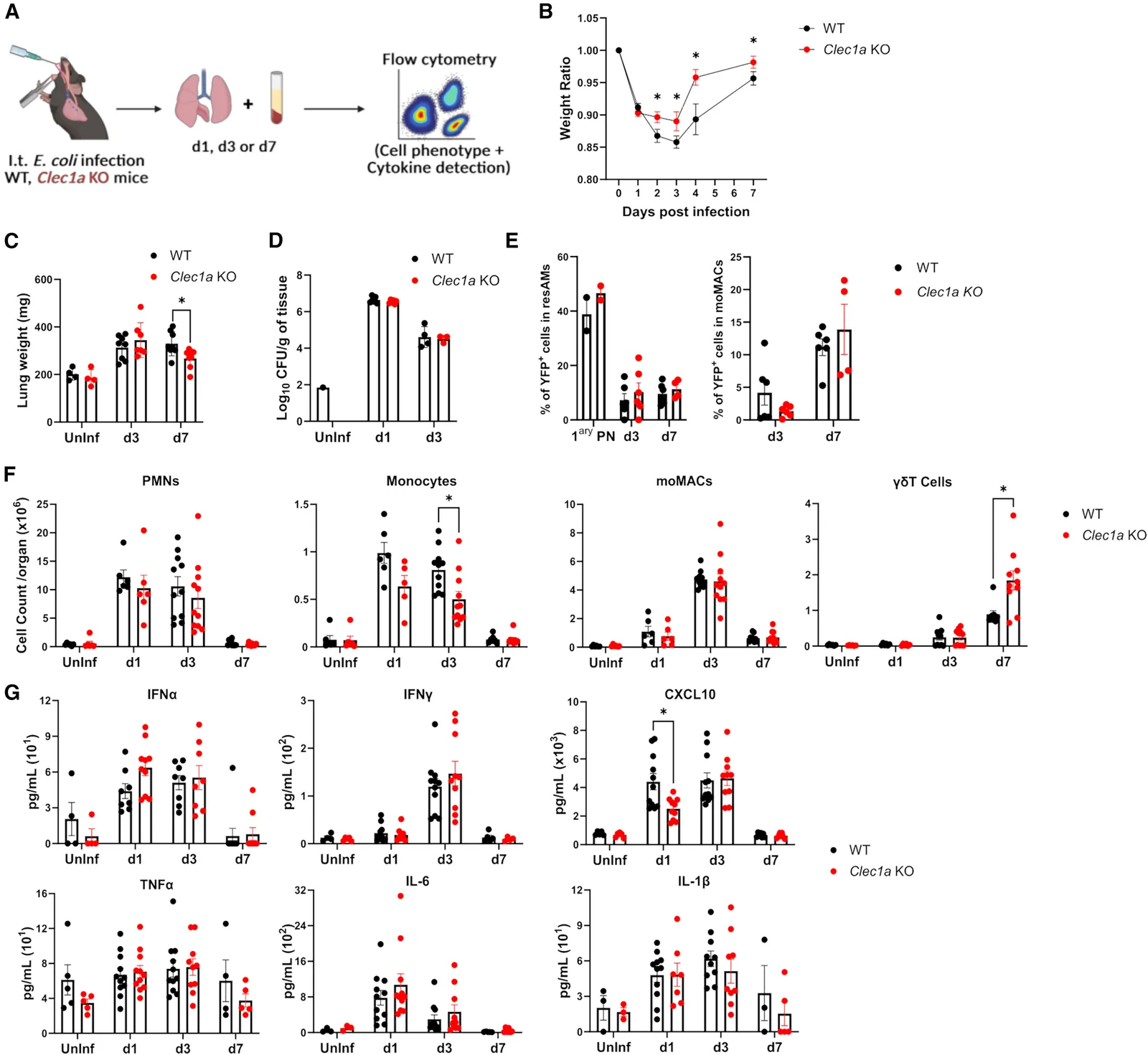
Absence of CLEC-1 improves mouse recovery and reduces CXCL10 and monocyte infiltration during sepsis (A) Workflow represents primary infection experiment for cell phenotype and cytokine detection in WT and *Clec1a* KO mice during sepsis. (B) Weight ratio (weight at day X/initial weight of the animal presented as mean ± SEM) over time of WT and *Clec1a* KO mice following intratracheal *E. coli* infection (75 μL of DH5α intratracheal, McF = 0.7) (For each genotype, *n* = 18 at day 0, *n* = 11 at day 1 and 2, *n* = 15 at day 3, *n* = 8 at day 4 and 7 from four independent experiments, ∗*p* < 0.05 by Mann-Whitney *t* test). (C) Lung weight of WT and *Clec1a* KO mice in uninfected condition (Uninf) and at day 3 and 7 after intratracheal *E. coli* infection presented as mean ± SEM (Uninf *n* = 4 for each genotype; d3 and 7 *n* = 8 for WT and 7 for *Clec1a* KO from two independent experiments, ∗*p* < 0.05 by Mann-Whitney *t* test). (D) Enumeration of colony-forming units (CFU) per gram of lung (CFU/g of tissue) analyzed in WT and *Clec1a* KO mice in uninfected condition (Uninf) and at day 1 or 3 after intratracheal *E. coli* infection and presented as mean ± SEM (Uninf *n* = 2 for WT; d1 *n* = 8 for WT and 6 for *Clec1a* KO; d3 *n* = 4 for WT and 3 for *Clec1a* KO from two independent experiments). (E*)* Percentages of resAMs and moMACs presented as mean ± SEM from WT and *Clec1a* KO mice having phagocytosed YFP-*E. coli* 2 h after infection (primary pneumonia (1^ary^ PN) or at day 3 and 7 after a primary pneumonia (1^ary^ PN *n* = 2 for WT; d3 *n* = 6 for each genotype; d7 *n* = 6 for WT and 4 for *Clec1a* KO from two independent experiments). (F) Flow cytometry cell count per lung of polymorphonuclear cells (PMNs), monocytes, monocyte-derived macrophages (moMACs) and γδ T cells from WT and *Clec1a* KO mice in uninfected condition (Uninf) or at day 1, 3 and 7 after intratracheal *E. coli* infection and presented as mean ± SEM (Uninf and d1 *n* = 6 for each genotype; d3 *n* = 11 for each genotype; d7 *n* = 9 for WT and 11 for *Clec1a* KO from two independent experiments, ∗*p* < 0.05 by Mann-Whitney *t* test). (G) Cytokines and chemokines were analyzed by flow cytometry using a multiplex bead-based assay from the sera of WT and *Clec1a* KO mice in uninfected condition (Uninf) and at day 1, 3 and 7 after intratracheal *E. coli* infection (Uninf *n* = 4–5; d1 *n* = 11; d3 *n* = 10; d7 *n* = 5 for each genotype from two independent experiments, ∗*p* < 0.05 by Mann-Whitney *t* test) and presented as mean ± SEM.

To assess the role of CLEC-1 in sepsis, we used C57BL/6 *Clec1a* knockout (KO) mice. We previously showed that these mice exhibit a normal immune cell composition and no obvious developmental abnormalities or signs of pathology at steady state.^14^ In these mice, sepsis was induced by using a well-established model of lung infection through intratracheal delivery of *E. coli* (Figure 1A), one of the most frequent bacteria responsible for hospital-acquired pneumonia. This mouse model closely mimics critically ill patients developing hospital-acquired pneumonia after systemic inflammation.^6^^,^^9^ This model of *E coli* infection is characterized by an acute phase from day 1–3 with profound weight loss, followed by a progressive recovery phase until the sepsis-cured state at day 7.^6^ Interestingly, we observed in this model that *Clec1a* KO mice exhibited a less severe body weight loss and a faster recovery than wild type (WT) mice following infection (Figure 1B). Moreover, lungs from *Clec1a* KO mice displayed a lower weight compared to the ones from WT mice at day 7, suggesting reduced edema and better recovery following infection (Figure 1C). We assessed if this faster recovery was due to a better bacterial clearance in the absence of CLEC-1. However, no differences were found in the lung bacterial burden between both genotypes at day 1 or 3 post-infection (Figure 1D). Moreover, no changes were noticed between both genotypes in the ability of resident alveolar macrophages (resAMs) defined as CD11c^-hi^, CD11b^-lo^, F4-80^+^, SiglecF^+^ cells or of monocyte derived macrophages (moMACs) defined as CD11c^-lo^, CD11b^-hi^, F4-80^+^ cells (gating strategy Figure S1A), to phagocytose yellow fluorescent bacteria (YFP+ *E. coli*) at day 3 and 7 post-infection or even during primary pneumonia (Figure 1E).

We then evaluated whether this better recovery observed with *Clec1a* loss was due to a modulation of the inflammatory response by analyzing, by flow cytometry, the number and phenotype of different immune cells from the lungs during the acute phase of sepsis and in sepsis-cured mice. In this model, *E coli* infection is characterized by a massive infiltration of the lung by neutrophils and monocytes, peaking on day 1 post-sepsis, followed by the infiltration of moMACs, monocyte-derived DCs (moDCs), natural killer (NK) cells, and CD4^+^ and CD8^+^ T cells, peaking at day 3 post-sepsis (gating strategy Figure S1A) (Figures 1F and S2A). Moreover, numerous γδ T cells expressing the marker of tissue residency CXCR6, accumulate in the lungs at day 7, once the mice have recovered from sepsis (Figure 1F). Interestingly, we observed a reduction of lung infiltration by monocytes in *Clec1a* KO mice compared to WT mice during the acute phase of sepsis and particularly at day 3 (Figure 1F). In contrast, no differences were noted in the total number of pulmonary resAMs, cDC1s or cDC2s nor in the number of infiltrating moDCs, moMACs, NK cells, CD4^+^ or CD8^+^ T cells at day 1, 3 or 7 post sepsis between the two genotypes (Figures 1F and S2A). Of note, we observed an increased accumulation of γδ T cells in *Clec1a* KO mice compared to WT mice at day 7 in sepsis-cured (Figure 1F).

We then assessed the expression levels of pro-inflammatory cytokines (TNFα, IL-6, IL-1β, IFNα, IFNγ) and chemokines (CXCL1, CCL2, CXCL10, CCL5/RANTES) in the lungs and the sera of infected mice. In the lungs, their levels were highly heterogeneous between samples, and we only observed a tendency for decreased levels of IL-6, CXCL10, and CCL2 in *Clec1a* KO mice compared to WT mice at day 1 or 3 post-sepsis (Figure S2C). In the sera, we detected a significant reduction in the level of CXCL10 in *Clec1a* KO mice compared to WT mice as soon as day 1 post-sepsis, whereas no difference was observed for the other cytokines and chemokines (Figures 1G and S2B). CXCL10, an IFN-induced chemokine predominantly expressed by myeloid cells, is involved in the recruitment of inflammatory cells such as monocytes and T cells, and has been reported to exacerbate tissue damage and sepsis pathology.^19^^,^^20^ We denoted no difference between the two genotypes in terms of IFNα levels, which, —unlike IFNγ levels—peak on the first day after sepsis. This suggests that the reduction in CXCL10 levels observed in *Clec1a* KO mice as soon as day 1 post-sepsis was not due to lower IFN levels, but could result from a decrease in the response of myeloid cells to type I IFN. Alternatively, this may reflect the lower influx of inflammatory monocytes later observed in the lungs.

Collectively, these results suggest that absence of CLEC-1 may dampen the excessive inflammatory response occurring during sepsis not by improving bacterial clearance, but rather by reducing the early recruitment of pro-inflammatory monocytes.

### Absence of CLEC-1 limits the early IFN response in lung cDC1s during sepsis

**Figure 2:**
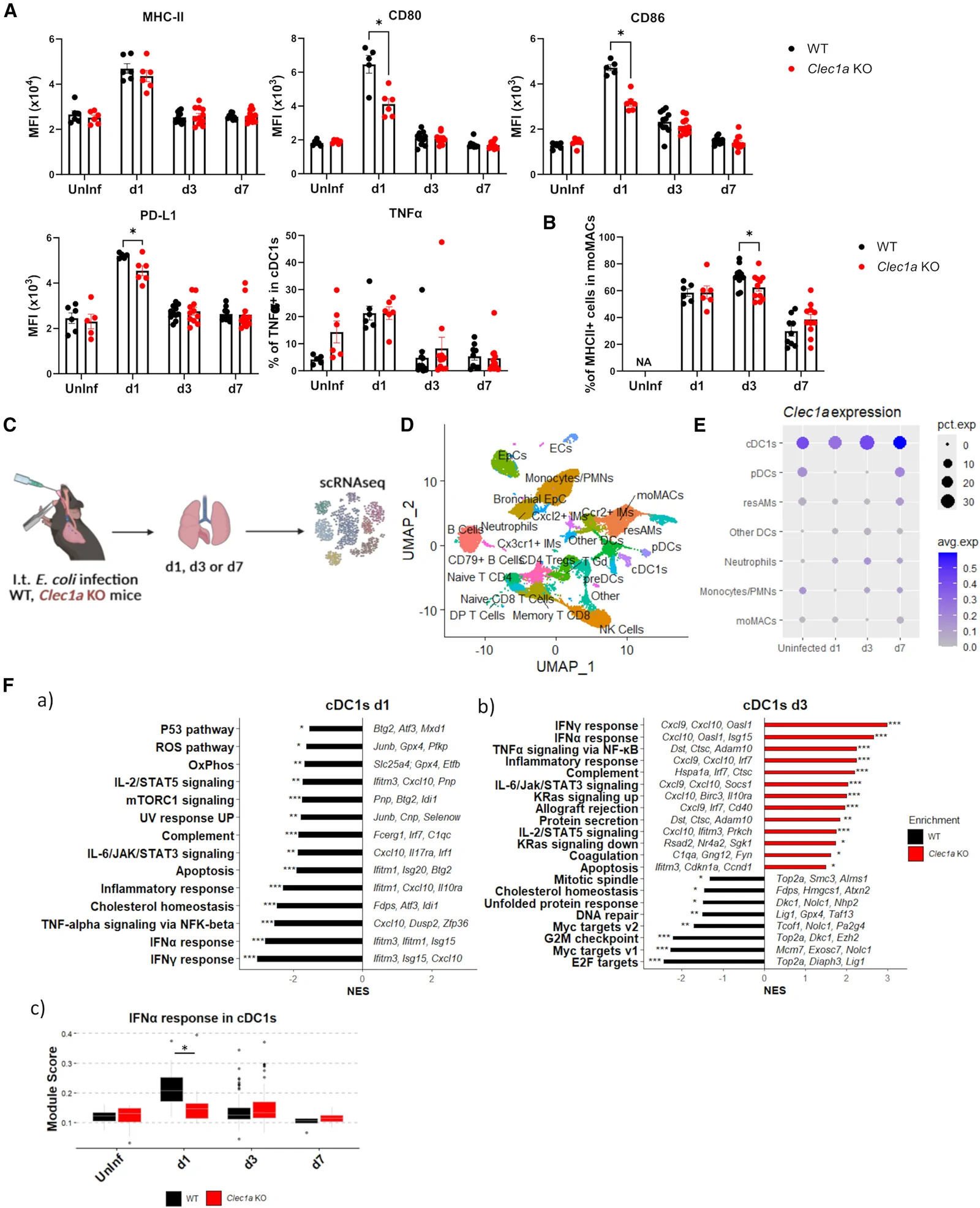
Absence of CLEC-1 limits the early IFN pathways in lung cDC1s during sepsis (A) Expression of activation markers on lung cDC1s from WT and *Clec1a* KO mice in uninfected condition and at day 1, 3 and 7 following intratracheal *E. coli* infection (Uninf *n* = 6; d1 *n* = 5–6; d3 *n* = 10–11; d7 *n* = 9–11 for each genotype from two independent experiments, ∗*p* < 0.05 by Mann-Whitney *t* test). Data were assessed by flow cytometry and represented by the Median Fluorescence Intensity (MFI) as mean ± SEM. (B) Expression of MHC-II in lung moMACs from WT and *Clec1a* KO mice in uninfected condition and at day 1, 3 and 7 following intratracheal *E. coli* infection (d1 *n* = 6; d3 *n* = 10–11; d7 *n* = 9–11 for each genotype from two independent experiments, ∗*p* < 0.05 by Mann-Whitney *t* test). Data were assessed by flow cytometry and represented by the percentage of MHC-II positive cells in moMACs as mean ± SEM. NA: not applicable. (C) Workflow represents primary infection experiment for scRNA-seq experiments in WT and *Clec1a* KO mice during sepsis. (D) Uniform manifold approximation and projection (UMAP) of cell populations in the lungs from WT and *Clec1a* KO mice following intratracheal *E. coli* infection. (E) *Clec1a* transcript expression obtained by scRNA-seq in several subpopulations of myeloid cells from WT and *Clec1a* KO mice in uninfected conditions and at days 1, 3, and 7 following intratracheal *E. coli* infection. The size of the dots (circles) indicates the percentage of cells expressing the gene, while the color of the dots indicates the average gene expression level. (F) Gene set enrichment analysis (GSEA) shows the Hallmark Pathways significantly enriched (a) at day 1 and (b) at day 3 and (c) module score of the Hallmark IFNα response pathway in the scRNA-seq cluster of lung cDC1s from WT and *Clec1a* KO mice in uninfected condition (UnInf) or following intratracheal *E. coli* infection. GSEA results represent the normalized enrichment score (NES), and the adjusted *p* value was estimated by an adaptive multi-level split Monte-Carlo scheme used by the fgsea package, ∗*p* < 0.05, ∗∗*p* < 0.01, and ∗∗∗*p* < 0.001. For D-F, cells were derived from *n* = 2 mice for uninfected, d1, and d7, and from *n* = 4 mice for d3 for each genotype.

We then analyzed, by flow cytometry, at days 1, 3, and 7 after sepsis, the expression of canonical activation markers in different subsets of resident and infiltrating myeloid cells from the lungs, such as DCs, monocytes, and macrophages. On day 1, the most striking effect of the absence of CLEC-1 was observed on the phenotype of cDC1s. We found that in contrast to cDC1s from WT mice, which strongly upregulate the expression of CD80, CD86, and PD-L1 costimulatory molecules at day 1 post-infection, their induction was significantly lower in cDC1s from *Clec1a* KO mice (Figure 2A). In contrast, no significant difference was noted in their expression of TNFα, suggesting that not all pro-inflammatory molecules were reduced in the absence of CLEC-1. Other tissue-resident populations, such as other cDC subtypes or resAMs, did not exhibit significant changes in their expression of activation markers during sepsis between both genotypes (Figures S3A and S3B). Regarding lung infiltrating cells, we did not find any difference in the percentage of monocytes expressing MHC-II between both genotypes (Figure S3C), but found a reduced proportion of recruited moMACs expressing MHC-II in *Clec1a* KO mice compared to WT mice at day 3 post-sepsis, suggesting a reduced pro-inflammatory state of these cells (Figure 2B).

To better compare the activation state between WT and *Clec1a* KO mice, we analyzed the transcriptomic profile of lung resident and infiltrating cells by single-cell RNA sequencing (scRNAseq) at day 1, 3 and 7 post sepsis (Figure 2C). Determination of cell type labels was done by a first step of unsupervised clustering, followed by cluster annotation based on canonical markers found in the differentially expressed genes of each cluster (Figures S1B and S1C). We identified several cell clusters, including myeloid cell subsets such resAMs, moMACs, neutrophils, monocytes/polymorphonuclears (PMNs), cDC1s, plasmacytoid DCs (pDCs), and other types of DCs (Figure 2D). Visualization of the different cell clusters during the disease course illustrates the massive infiltration by monocytes/PMNs and moMACs, which culminate at day 3 post-sepsis (Figure S1B).

In this scRNA-seq dataset, we first analyzed the expression of *Clec1a* transcripts during the disease course. We noted that lung cDC1s were the myeloid cells exhibiting the highest level of *Clec1a* transcripts during sepsis (Figure 2E). In these cells, the amount of *Clec1a* transcripts appeared to decrease at day 1 post-sepsis, which corresponds to the peak of the pro-inflammatory phase, and then to increase again from days 3–7 during the resolution phase. This regulation of *Clec1a* expression is in accordance with what we previously showed in DCs, with particular expression by the cDC1 subset and its downregulation by pro-inflammatory stimuli such as LPS.^14^^,^^15^^,^^16^ Of note, although expressed at a much lower level than in cDC1s, *Clec1a* transcripts in pDCs, resAMs, neutrophils, monocytes/PMNs, and moMACs appeared to increase progressively during sepsis. In this scRNA-seq dataset, we then compared, by Gene Set Enrichment Analysis (GSEA), the transcripts of different cell subsets from WT and *Clec1a* KO mice at different time points after sepsis. No significant difference was observed between WT and *Clec1a* KO cell subsets at steady state (data not shown). However, at day 1 post-sepsis, we found that gene sets associated with the pathways of IFNγ and α responses were significantly decreased in cDC1s from *Clec1a* KO compared to WT mice (Figure 2Fa). This was no longer observed at the end of the acute phase at day 3, where enrichment of these pathways was even reversed and appeared upregulated in cDC1s from *Clec1a* KO compared to WT mice (Figure 2Fb). To illustrate the kinetics over time of the IFN response pathway in cDC1s, we performed a boxplot of the module activation score at day 1, 3, and 7 after sepsis. We noticed that in contrast to cDC1s from WT mice that expressed a high module score of the IFNα response pathway peaking at day 1 post-sepsis, cDC1s from *Clec1a* KO mice expressed a lower score at this time point (Figure 2Fc). In contrast, the IFNα response, which is later strongly reduced at day 3 post-sepsis in WT mice, is less decreased in *Clec1a* KO mice, suggesting a reduced counter-regulation of this pathway following the peak in the absence of CLEC-1. Such transcript dysregulation of the IFN pathways was not observed in other cDC subsets, pDCs, or endothelial cells between WT and *Clec1a* KO mice at different time points after sepsis, suggesting that it was restricted to cDC1s (data not shown).

However, at day 3, which corresponds to the end of the acute phase of sepsis, we observed that the moMACs which massively infiltrate the lungs at this time point and that we found to expressed lower levels of MHC-II (Figure 2B), exhibited a decreased enrichment for gene sets associated with TNFα signaling and pro-inflammatory response in *Clec1a* KO mice compared to WT mice (Figure S3D). In contrast, their gene sets associated with IFNα response and allograft rejection, including antigen-presenting molecules, were increased at this time point, suggesting that these cells may be more prone to present antigens and mount a secondary antibacterial IFN response at the end of the acute phase.

Altogether, these data suggest that absence of CLEC-1 reduces the IFN response in lung cDC1s at the early onset of sepsis. Consequently, this may limit the pro-inflammatory state of the recruited moMACs, dampening persistent inflammation and immunopathology.

### Absence of CLEC-1 reduces the immunosuppressive state of lung myeloid cells following secondary infection

**Figure 3:**
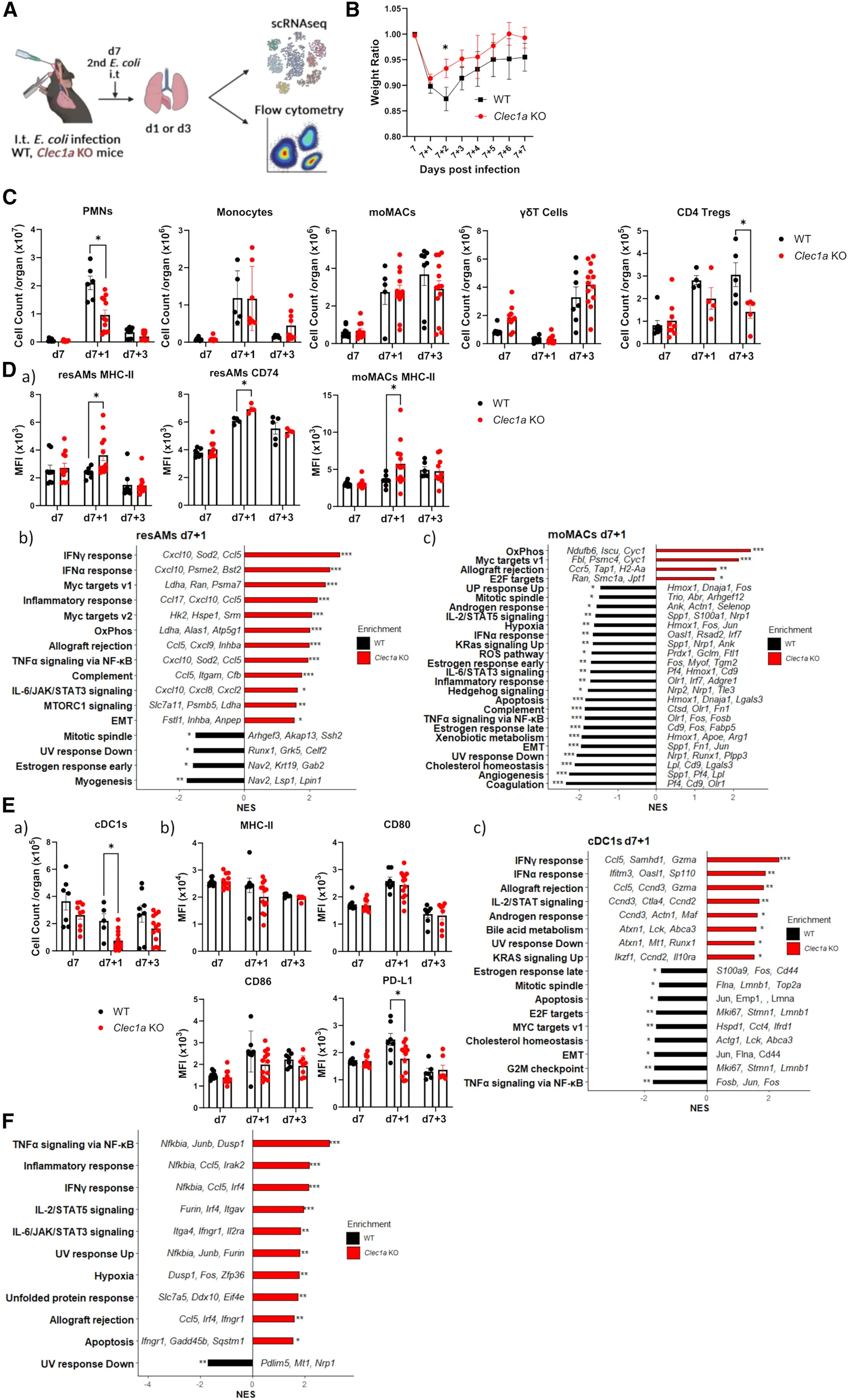
Absence of CLEC-1 reduces the immunosuppressive phenotype of lung myeloid cells following secondary *E. coli* infection (A) Workflow representing secondary infection experiment for cell phenotype and scRNA-seq experiments in WT and *Clec1a* KO mice during sepsis. (B) Weight ratio (weight at day X/initial weight of the animal presented as mean ± SEM) over time following secondary intratracheal *E. coli* infection (75 μL of DH5α intratracheal, McF = 0.7) performed in WT and *Clec1a* KO mice at day 7 after primary infection (d7 and d(7 + 1) *n* = 9 for WT and 22 for *Clec1a* KO; d(7 + 2) and d(7 + 3) *n* = 6 for WT and 13 for *Clec1a* KO; d(7 + 4) to d(7 + 7) *n* = 3 for WT and 5 for *Clec1a* KO from four independent experiments, ∗*p* < 0.05 by Mann-Whitney *t* test). (C) Flow cytometry cell count per lung of different immune cell populations in the lungs from WT and *Clec1a* KO mice at day 7 after primary infection and at day 1 (7 + 1) and 3 (7 + 3) following secondary intratracheal *E. coli* infection (*n* = 7–9 for WT and 13–15 for *Clec1a* KO from two independent experiments, ∗*p* < 0.05 by Mann-Whitney *t* test) and presented as mean ± SEM. (D) a) Expression of MHC-II and CD74 on lung resAMs and moMACs at day 7 after primary infection and at day 1 (7 + 1) and 3 (7 + 3) following secondary intratracheal *E. coli* infection (*n* = 6–8 for WT and 12–13 for *Clec1a* KO from two independent experiments, ∗*p* < 0.05 by Mann-Whitney *t* test) assessed by flow cytometry and represented by the Median Fluorescence Intensity as mean ± SEM. b) Gene set enrichment analysis (GSEA) showing the Hallmark Pathways significantly enriched in the scRNA-seq cluster of lungs resAMs and c) moMACs from WT and *Clec1a* KO mice at day 1 following secondary infection. (E) a) Flow cytometry cell count per lung and b) expression of activation markers of cDC1s from the lungs of WT and *Clec1a* KO mice at day 7 after primary infection and at day 1 (7 + 1) and 3 (7 + 3) following secondary *E. coli* infection (*n* = 5–8 for WT and 9–13 for *Clec1a* KO from two independent experiments, ∗*p* < 0.05 by Mann-Whitney *t* test) assessed by flow cytometry and represented by the Median Fluorescence Intensity (MFI) as mean ± SEM. c) Gene set enrichment analysis (GSEA) showing the Hallmark Pathways significantly enriched in the scRNA-seq cluster of lung cDC1s from WT and *Clec1a* KO mice at day 1 following secondary infection. (F) Gene set enrichment analysis (GSEA) showing the Hallmark Pathways significantly enriched in the scRNA-seq cluster of lung γδ T cells from WT and *Clec1a* KO mice at day 1 following secondary infection. For images with GSEA, results represent the normalized enrichment score (NES), and the adjusted *p* value was estimated by an adaptive multi-level split Monte-Carlo scheme used by the fgsea package, ∗*p* < 0.05, ∗∗*p* < 0.01, and ∗∗∗*p* < 0.001. For all images, data are representative of at least two independent experiments.

Major hallmarks of sepsis-induced immunosuppression include lymphocyte apoptosis, the exhaustion of monocyte/macrophage, and the accumulation in the lungs of immunosuppressive cells such as polymorphonuclear myeloid-derived suppressor cells (PMNs-MDSCs) and Tregs.^5^ Moreover, lung DCs and macrophages express inhibitory immune checkpoints such as PD-L1 and are unable to present antigens and mount an efficient secondary immune response.^5^^,^^9^ Therefore, in order to determine the implication of CLEC-1 in the dysregulation of immune response and long-term immunosuppression, we rechallenged the sepsis-cured mice by intratracheal *E. coli* infection at day 7 (Figure 3A).

As for primary infection, we observed reduced weight loss in *Clec1a* KO mice compared to WT mice during the secondary challenge with the pathogen, suggesting less immunopathology in the absence of CLEC-1 (Figure 3B). We then assessed the number and phenotype of the immune cells from the lung over time. Contrasting with primary infection, we found a significant decrease in the early accumulation of PMNs at day 1 following the secondary challenge in *Clec1a* KO mice compared to WT mice, whereas no difference was observed for monocytes or macrophages (Figure 3C). Interestingly, we denoted a significant decrease of the accumulation of Foxp3 Tregs in the lungs of *Clec1a* KO mice compared to WT mice at day 3 upon the secondary challenge whereas the number of γδ T cells tended to increase as for primary infection (Figure 3C). Regarding the phenotype, we found in the absence of CLEC-1 that lung resAMs and moMACs displayed an increased expression of the MHC-II and/or CD74 antigen presentation molecules as soon as the first day upon secondary challenge (Figure 3Da). In addition, scRNA-seq transcriptomic analysis revealed that these cells displayed an upregulated enrichment of pathways related to allograft rejection on day 1 upon secondary challenge. This was characterized by their up-regulation of genes associated with better antigen presentation, such as *Tap1* and *H2-Aa* (Figures 3Db-c). Furthermore, as for primary infection, moMACs exhibited reduced expression of pathways involved in the pro-inflammatory response in *Clec1a* KO mice compared to WT mice (Figure 3Dc).

Regarding lung cDC1s, we noted a drop in their number in *Clec1a* KO mice compared to WT mice upon secondary challenge, and as for primary infection, these cells expressed a lower level of the immune checkpoint PD-L1 (Figure 3Ea-b). However, contrasting with primary infection, scRNA-seq analysis revealed that following secondary infection, these cells were already enriched from day 1 with gene sets associated with stimulatory properties such as IFN responses and allograft rejection compared to ones from WT mice (Figure 3Ec)*.* Moreover, we observed that the γδ T cells, which strongly accumulated in the lung during sepsis, exhibited at their transcriptomic level an enrichment of gene sets associated with inflammatory and IFNγ responses, suggesting a more activated profile (Figure 3F). No major differences were found in the proteomic and transcriptomic profiles of the other identified cell subsets (data not shown).

Overall, these data reveal a better stimulatory profile of macrophages and DCs and a decreased accumulation of Tregs in the lung following secondary infection in the absence of CLEC-1, suggesting reduced hallmarks of immunosuppression.

### Absence of CLEC-1 negatively regulates some pathways of the IFN response in cDC1s

We investigated whether the down-regulation of the IFN pathways observed at the early time point of sepsis in lung cDC1s from *Clec1a* KO mice was due to an intrinsic role of CLEC-1 in these cells. By exploiting the scRNA-seq data, we did not notice any difference in the expression of the IFN receptors *Ifnar1/2* and *Ifngr1/2* between lung cDC1s from WT and *Clec1a* KO mice at steady state or at different time points after sepsis (Figure S4). This suggests that this differential IFN response observed in lung cDC1s between both genotypes was not due to a difference in their IFN receptor expression. We then sought to determine whether this was due to regulation of IFN production by these cells or to regulation of the IFN signaling pathway. Unfortunately, due to technical limitations of the scRNA-seq technique, we were not able to detect type I IFN transcripts in lung cDC1s at steady state or during sepsis.

**Figure 4:**
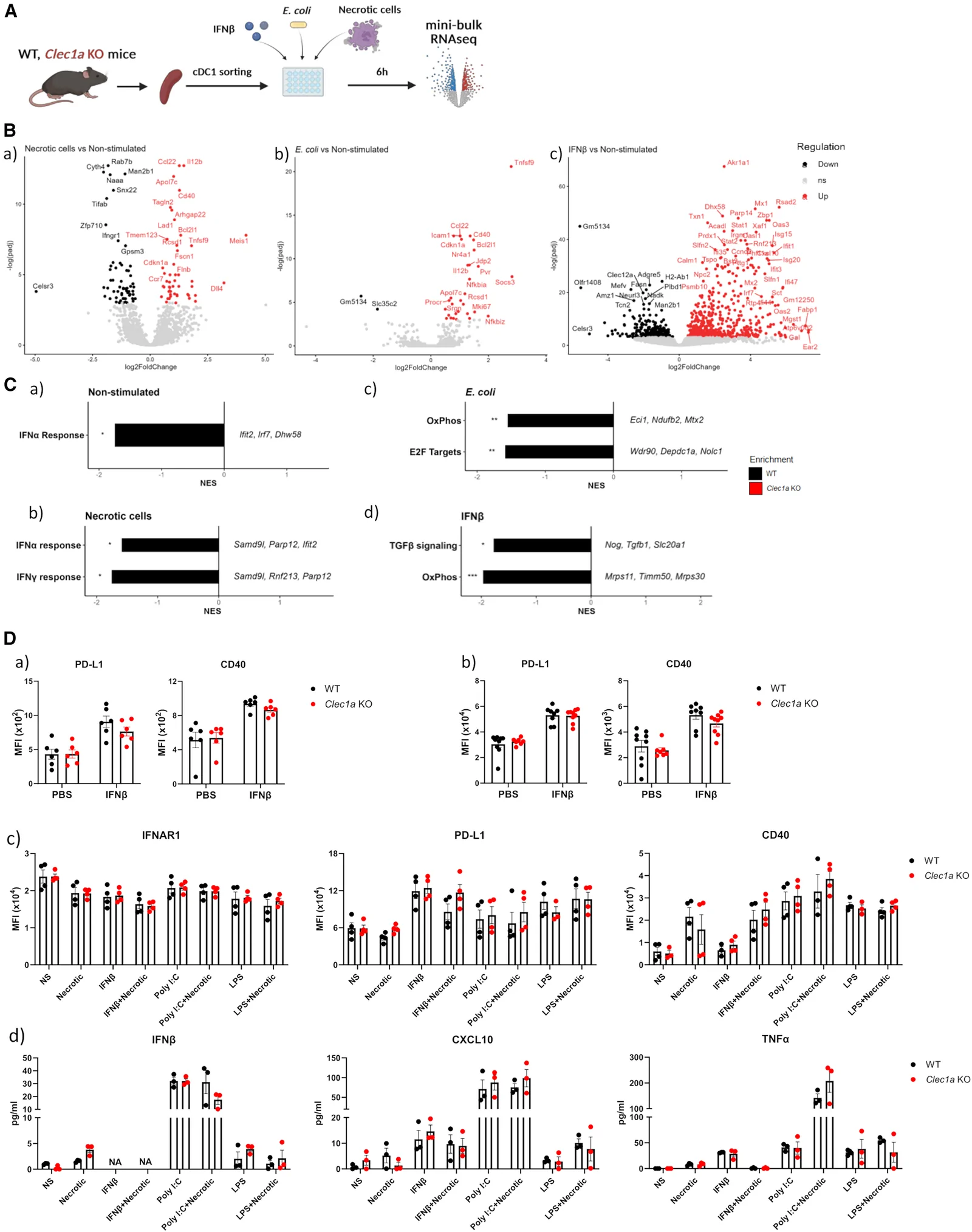
Purified cDC1s from *Clec1a* KO mice downregulate some IFN signaling pathways in response to necrotic cells (A) Workflow representing the mini-bulk RNA-seq experiments on purified splenic cDC1s from WT and *Clec1a* KO mice. (B) Volcano plots obtained by mini-bulk RNA-seq illustrating upregulated and downregulated genes in purified splenic cDC1s from WT mice stimulated *in vitro* with a) necrotic cells (ratio 1:10), b) *E. coli* (MOI = 10:1), or c) recombinant mouse IFNβ (1000 U/ml) for 6 h and compared versus non-stimulated (*n* = 6 mice in 2 independent experiments). Data represent the log2(FoldChange) versus the -log(Adjusted pValue) with a padj cutoff of 0.05, where the most significant genes were labeled. (C) Gene set enrichment analysis (GSEA) showing the Hallmark Pathways significantly enriched in purified splenic cDC1s from *Clec1a* KO mice versus WT mice in a) non-stimulated condition or following *in vitro* stimulation with b) necrotic cells (ratio 1:10), c) *E. coli* (MOI = 10:1) or d) recombinant mouse IFNβ (1000 U/ml) for 6 h. The intensity of the pathway enrichment in *Clec1a* KO mice versus WT mice is represented by the normalized enrichment score (NES), and the adjusted *p* value was estimated by an adaptive multilevel split Monte-Carlo scheme used by the fgsea package; ∗*p* < 0.05, ∗∗*p* < 0.01, and ∗∗∗*p* < 0.001. (D) Expression of activation markers evaluated by flow cytometry on cDC1s from the spleen of WT and *Clec1a* KO mice a) harvested 17h after *in vivo* intravenous injection of 30000 IU of mouse recombinant IFNβ (*n* = 6 mice per genotype and timepoint from 2 independent experiments) or b) stimulated *ex vivo* with 3000 IU/mL of mouse recombinant IFNβ for 17 h (*n* = 9 mice per genotype and timepoint from 3 independent experiments) or c) and d) stimulated *in vitro* (following purification) for 17 h with necrotic cells (ratio 1:10) alone or together with recombinant mouse IFNβ (1000 U/ml), Poly (I:C) (TLR3-L, 20 μg/mL) or LPS (TLR4-L, 0.1 μg/mL) (*n* = 4 mice for phenotype (c) and *n* = 3 mice for supernatants (d) per condition from 2 independent experiments) (NA: not applicable). Then, cells were extensively washed, harvested, and analyzed by RNA sequencing (RNAseq), or flow cytometry for phenotype, and cytokines and chemokines were analyzed by flow cytometry using a multiplex bead-based assay (data were represented by the Median Fluorescence Intensity (MFI) for phenotype in pg/ml for cytokines and chemokines and presented as mean ± SEM).

We therefore tried to figure out whether CLEC-1 plays an intrinsic role in DC1s in type I IFN signaling in response to DAMPs, PAMPs or in response to type I IFN per se. To this purpose, we examined the intrinsic role of CLEC-1 in cDC1 activation and IFNβ response at the transcriptomic level by bulk RNA-seq following *in vitro* incubation with necrotic cells, with *E coli,* or with recombinant IFNβ (Figure 4A). Due to the scarcity of pulmonary cDC1s, we used cDC1s purified from the spleen in order to obtain a sufficient number of cells. As illustrated by volcano plots, we observed that stimulation with necrotic cells, *E. coli* or IFNβ induced the up-regulation of numerous immune-related (*Cd40*, *Il12b,* or *Ccl22*) or IFN-induced (*Irf7*, *Isg15*, *Ifit1*) genes in cDC1s from WT mice (Figure 4Ba–c). When we compared by gene set enrichment analysis (GSEA) with cDC1s from *Clec1a* KO mice, we observed that some genes dependent on the IFN response pathways were down-regulated in cDC1s from *Clec1a* KO mice compared to WT mice in response to necrotic cells (Figure 4Cb). As this was already observed in non-stimulated conditions (Figure 4Ca), this suggests that ligands released by dying cDC1s during the assay may also have induced the negative regulation of this signaling pathway. In contrast, when the cDC1s were incubated with *E. coli* or recombinant IFNβ, no difference in the IFN pathways was observed between both genotypes, demonstrating that this was specific to necrotic cell stimulation. Nevertheless, cDC1s from *Clec1a* KO mice displayed a downregulation of oxidative phosphorylation, of the 2 EF targets, and of the TGFβ signaling pathways (Figure 4Cc-d), all known to favor immunosuppressive properties in DCs^21^^,^^22^^,^^23^ suggesting a role for CLEC-1 in immunosuppression following pro-inflammatory stimulation. At the protein level, we confirmed that cDC1s from *Clec1a* KO mice exhibit no defect in IFN signaling per se in response to recombinant IFNβ, as no significant difference between both genotypes was observed in the induction of type I IFN-induced molecules such as PD-L1 and CD40 following *in vivo*, *ex vivo,* and *in vitro* stimulation by recombinant IFNβ (Figure 4Da-c). No difference was observed between the two genotypes for IFNAR1 expression by purified cDC1s or for their production of TNFα, IFNβ and CXCL10 following *in vitro* stimulation with recombinant IFNβ, TLR4-L (LPS), or TLR3-L (Poly (I:C)), in combination or not with necrotic cells (Figure 4Dc-d). These data suggest that CLEC-1 does not play an intrinsic role, at least *in vitro*, in the maturation process of cDC1s involving IRF3 or the NF-κB pathway.

Taken together, these *in vitro* experiments reveal a differential regulation of some IFN pathways in purified cDC1s in the absence of CLEC-1 that is not due to a loss of IFNAR expression, or to defective maturation, IFN production, or IFNβ signaling per se.

### Anti-human CLEC-1 antagonist mAb attenuates the pro-inflammatory response in CLEC-1 humanized mice during sepsis

**Figure 5:**
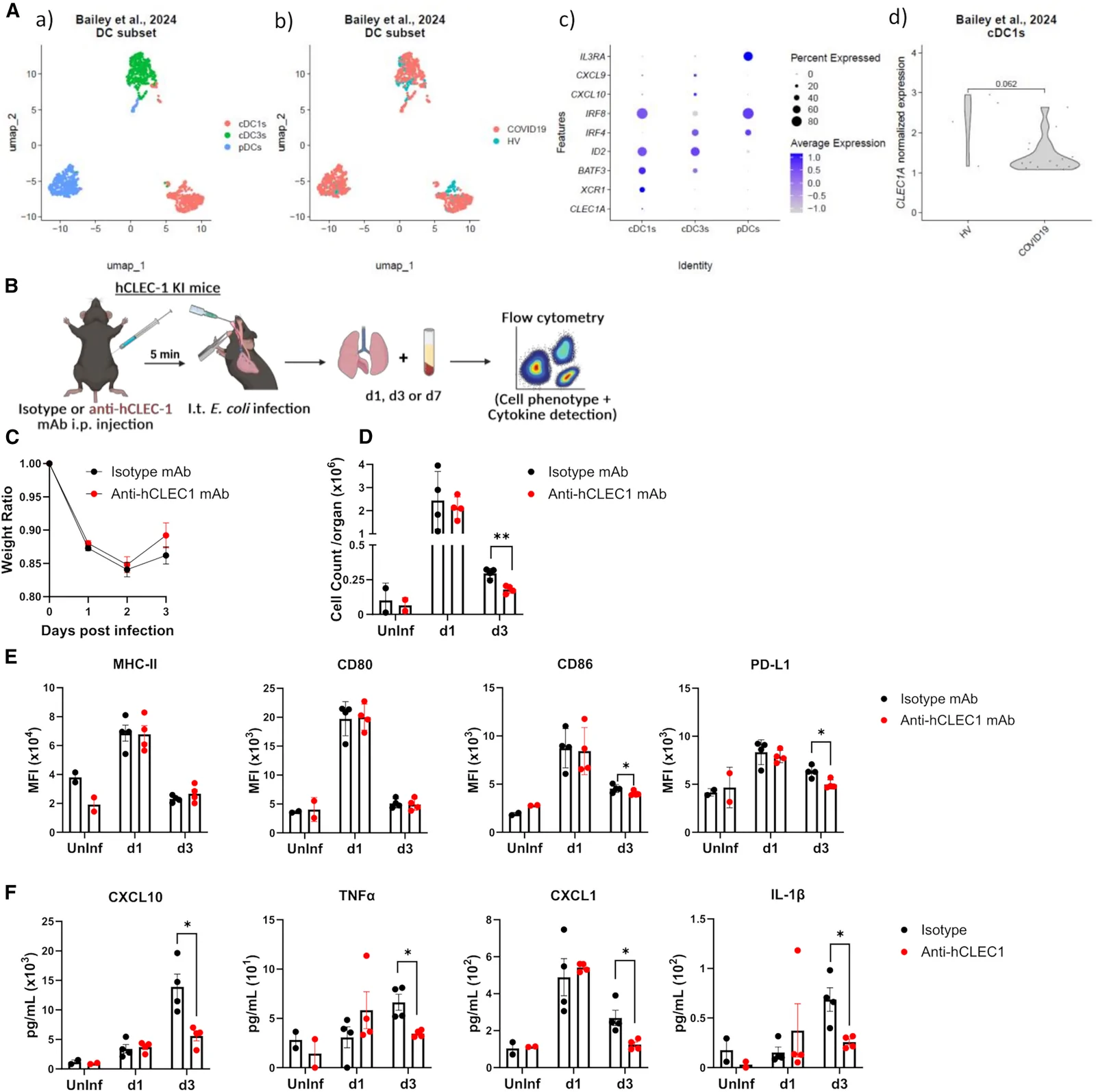
Clinical relevance and therapeutic potential of CLEC-1 blockade with a mAb during sepsis (A) scRNA-seq analysis from a public dataset of human lung cDC subsets from healthy volunteers (HV) and patients with COVID-19. a) Visualization on a Uniform Manifold Approximation and Projection (UMAP) plot obtained with the Seurat R package illustrates the different cDC clusters and b) among healthy volunteers (HV) and patients with COVID-19. Cells are colored based on cell type annotations, as indicated in the legend. c) Representative marker genes and *CLEC1A* expression in the human DC subclusters. The size of the dots (circles) indicates the percentage of cells expressing the gene, while the color of the dots indicates the average gene expression level. d) *CLEC1A* normalized expression between healthy volunteers (HV) and patients with COVID-19. (B) Workflow represents primary infection experiment for cell phenotype and cytokine detection following CLEC-1 neutralization in hCLEC-1 KI mice during sepsis. (C) Weight ratio (weight at day X/initial weight of the animal presented as mean ± SEM) over time of hCLEC-1 KI mice following intratracheal *E. coli* infection (75 μL of DH5α intratracheal, McF = 0.7) and intraperitoneal injection of 100 μg of anti-hCLEC-1 or its corresponding isotype control (*n* = 8 at day 0 and 1 and *n* = 4 at day 2 and 3 from two independent experiments for isotype and anti-hCLEC-1 mAb treated mice). (D) Flow cytometry cell count per lung of monocytes from the lungs of hCLEC-1 KI mice treated intraperitoneally with either 100 μg of an anti-hCLEC-1 antagonist monoclonal antibody (mAb) or 100 μg of its corresponding isotype in uninfected condition (Uninf) and at day 1 and 3 after *E. coli* infection and presented as mean ± SEM (*n* = 2 in Uninf and *n* = 4 at d1 and 3 for isotype and anti-hCLEC-1 mAb treated mice, ∗*p* < 0.05 by Mann-Whitney *t* test). (E) Expression of activation markers of cDC1s from the lungs of hCLEC-1 KI mice treated intraperitoneally with either 100 μg of an anti-hCLEC-1 antagonist monoclonal antibody (mAb) or 100 μg of its corresponding isotype in uninfected condition (Uninf) and at day 1 and 3 after *E. coli* infection (*n* = 2 in Uninf and *n* = 4 at d1 and 3 isotype and anti-hCLEC-1 mAb treated-mice, ∗*p* < 0.05 by Mann-Whitney *t* test). Data were assessed by flow cytometry and represented by the Median Fluorescence Intensity (MFI) as mean ± SEM. (F) Cytokines and chemokines were analyzed by flow cytometry using a multiplex bead-based assay from the sera of hCLEC-1 KI mice treated intraperitoneally with either 100 μg of an anti-hCLEC-1 antagonist monoclonal antibody (mAb) or 100 μg of its corresponding isotype in uninfected condition (Uninf) and at day 1 and 3 after *E. coli* infection (*n* = 2 in Uninf and *n* = 4 at d1 and 3 for isotype and anti-hCLEC-1 mAb treated-mice, ∗*p* < 0.05 by Mann-Whitney *t* test) and were presented as mean ± SEM.

To evaluate the clinical relevance of CLEC-1 blockade to restore lung homeostasis in patients suffering from pneumonia, we analyzed public scRNA-seq datasets from bronchoalveolar lavage (BAL) of patients harboring persistent multi-organ symptoms after severe acute respiratory syndrome coronavirus-2 infection.^24^ We noticed that among the DC subsets identified in the BAL, *CLEC1A* transcripts were retrieved specifically in the cDC1 subset identified by *XCR1* and *BATF3* expression, corroborating the profile of expression observed in mice (Figure 5Aa-c). Moreover, we noted that its expression seems to be downregulated in cDC1s from the BAL of patients harboring persistent respiratory symptoms after coronavirus disease 2019 (COVID19) compared to healthy volunteers (Figure 5Ad). As long COVID19 is characterized by prolonged lung inflammation, these data suggest that the expression of *CLEC1A* by lung cDC1s in humans is downregulated in a pro-inflammatory environment, as observed in mice during sepsis (Figure 2E) but this will remain to be clearly established in patients with sepsis.

As proof of principle of the immunotherapeutic potential of CLEC-1 targeting during infection, we evaluated the effect of CLEC-1 blockade with a humanized anti-human CLEC-1 antagonist monoclonal antibody (mAb). We previously showed that this mAb blocks the interaction of CLEC-1 with necrotic cells.^14^ We tested the effect of this mAb administered in a single dose concomitantly with *E. coli* infection in humanized CLEC-1 knock-in (KI) mice (Figure 5B). These mice harbor the extracellular domain of mouse CLEC-1 replaced by its human counterpart, while the transmembrane and intracellular domains remain intact.^14^ Knowing the cross-reactivity of the ligands, these mice offer the advantage of harboring a fully functional immune system to evaluate the potential of CLEC-1 blockade with this mAb during sepsis. We did not observe a significant difference on body weight loss or on recovery following CLEC-1 signaling blockade with the mAb compared to control isotype (Figure 5C). However, by flow cytometry we found a reduced infiltration by monocytes at the end of the acute phase at day 3 of sepsis (Figure 5D). We also observed that cDC1s expressed lower levels of CD86 and PD-L1 costimulation molecules at day 3 post sepsis (Figure 5E). In addition, a lower level of CXCL10 but also of TNFα, CXCL1 and IL-1β was quantified in the sera of the mice at this time-point following CLEC-1 signaling blockade with the mAb (Figure 5F). These data suggest that CLEC-1 signaling blockade with the mAb recapitulated the reduction of the excessive inflammation observed by genetic ablation of *Clec1a.*

Collectively, these data suggest the therapeutic potential of CLEC-1 blockade with an anti-human CLEC-1 antagonist mAb to temper excessive inflammation during sepsis.

## Discussion

During sepsis, excessive pro-inflammatory response elicits dysregulation of signaling pathways, promoting an immunosuppressive environment, increasing susceptibility to secondary infections and subsequent mortality. The immunosuppressive response occurs as early as the inflammatory stage and is characterized by the expression of inhibitory immune checkpoints and the recruitment of immunosuppressive cells.^4^^,^^5^ cDC1s, which represent major actors of the immune response during pulmonary infection,^25^^,^^26^ acquire an immunoparalyzed phenotype contributing to Treg expansion and long-term immunosuppression.^6^^,^^9^ Therefore, these immunoregulatory pathways represent potential therapeutic targets for the management of patients with sepsis. In this study, we investigated, in an infectious context, the role of the innate receptor CLEC-1, which we had previously shown to act as an immune checkpoint in response to tissue damage in mice.^14^^,^^18^

Unexpectedly, we uncovered that *Clec1a* deficiency reduces weight loss, CXCL10 expression, and monocyte infiltration in a mouse model of sepsis. We found that the loss of *Clec1a* downregulates IFN pathways in pulmonary cDC1s at the onset of sepsis. Moreover, we assessed in the sera a reduced production of the IFN-induced CXCL10 chemokine, largely expressed by myeloid cells such as cDC1s. However, no difference was observed for the serum levels of type I or II IFNs. This suggests that the decrease in CXCL10 observed in the serum of *Clec1a* KO mice may be due to impaired IFN signaling rather than reduced IFN production following infection. Alternatively, this may reflect the reduced recruitment of pro-inflammatory monocytes observed in the lungs during the acute phase of sepsis. In the absence of CLEC-1, limited recruitment of pro-inflammatory monocytes may have attenuated immunopathology and improved mouse recovery at the end of the acute phase of sepsis. Similarly, we previously reported a reduction of monocyte/macrophage recruitment in *in vivo* models of sterile inflammation induced by tissue damage.^14^^,^^18^ We previously showed that this was corroborated with a reduced level of the chemoattractant CCL2. As CCL2 and CXCL10 are induced by type I IFN,^27^^,^^28^^,^^29^ a hypothesis could be that the limited IFN response by pulmonary cells such as resident cDC1s at the early step of sepsis may have reduced CXCL10 and CCL2 expression and downstream inflammatory monocyte recruitment, thereby reducing immunopathology and favoring mouse recovery.

*In vitro* experiments on purified cDC1s suggest an intrinsic role of CLEC-1 in the negative regulation of some IFN response genes. We showed that this regulation was not due to a defect in IFNAR expression or to IFNβ response per se. Moreover, this was not observed in response to *E. coli* or to TLR4 and TLR3 stimulation, supporting that regulation of this pathway may be specific to CLEC-1 activation by its endogenous ligand(s) on necrotic cells. Nevertheless, *in vitro* experiments may not reflect the plethora of signaling induced by DAMPs and PAMPs following *in vivo* infection, and it will remain to be elucidated if CLEC-1 plays a role in type I IFN production or IFN signaling in the specific response to necrotic cells and infection *in vivo*. Intriguingly, we previously identified the E3 ubiquitin ligase TRIM21 as an interacting partner of CLEC-1.^14^ TRIM21 is an intracellular protein induced by IFN that plays a critical role in the ubiquitination of proteins such as IFN regulatory factors, reinforcing a possible molecular interplay between CLEC-1 and the IFN response.^30^ Short-term inhibition of the IFN response at the onset of sepsis in lung-resident cDC1s may be important to prevent immune dysregulation. A deleterious effect of IFNβ during the acute phase of sepsis has been reported in some studies, and anti-IFNAR antibody treatment has been shown to reduce hyperinflammation and sepsis symptoms in mouse models.^31^ In contrast, during the recovery phase, IFN signaling has been shown to be crucial in infiltrating myeloid cells to prevent myeloid cell immunosuppression and inefficient response following secondary encounters with pathogens.^32^ These studies highlight the complexity of the IFN response, whose timing, location, nature of producer and responder cells, dose, and duration could be key in determining beneficial or harmful effects and disease outcomes. Of note, although endothelial cells also express *Clec1a*, we did not observe any regulation of the IFN pathways in these cells in the absence of CLEC-1 during sepsis, suggesting an effect restricted to cDC1s.

Lung resident cDC1s express a unique tissue-specific gene program, governing tolerogenic function to maintain lung homeostasis,^33^ but also serve as sentinels to protect from infection.^25^^,^^26^ We observed an early decrease in IFN signaling and PD-L1 expression in pulmonary cDC1s from *Clec1a* KO mice during sepsis. This early limitation of type I IFN response may be important to prevent monocyte recruitment and persistent inflammation but also to restrain immunosuppression. Indeed, type I IFN plays a dual role by providing necessary inflammatory signals but also by initiating feedback suppression, such as induction of PD-L1 expression. In sepsis, PD-L1 is involved in immune exhaustion and was found to be highly expressed in antigen-presenting cells in postmortem patients with sepsis. Its blockade has been shown to restore myeloid phagocytic function *in vitro* and prevent mortality *in vivo.*^34^ Moreover, type I IFN has been reported to exert immunosuppressive effects in DCs by inducing IL-10 expression and inhibiting IL-12 production and subsequent Th1 response.^35^^,^^36^^,^^37^^,^^38^ In this sense, type I IFN in response to lymphocytic choriomeningitis virus infection was found to suppress IL-12 production in mice, and administration of neutralizing IFNαβ Abs resulted in detectable levels of IL-12 and IFNγ.^39^ In our context, we did not observe any changes in the absence of CLEC-1 in the number of conventional T cells or in their IFNγ secretion following sepsis. However, we found more tissue-resident unconventional γδ T cells, and these cells exhibited a better effector profile. These γδ T cells were shown to be mostly IL-17^+^RORγt^+^, to derive from local progenitors, and to accumulate in the lungs during sepsis.^40^ They were shown to be critical to the overall protection of the host against pathogenic insults, as their deficiency contributes to the high mortality rate seen in many different models of bacterial infection.^41^ It remains to be elucidated whether the absence of CLEC-1 indirectly promotes proliferation of these cells or inhibits their tissue egress. Interestingly, we observed a reduced immunoparalyzed profile of pulmonary DCs and macrophages following secondary infection in the absence of CLEC-1, with a higher expression of antigen-presentation molecules. This was associated with a lower accumulation of Tregs in the lung. This suggests that this reduced pool of Tregs may have locally facilitated the maturation of myeloid cells. Alternatively, more mature myeloid cells may have prevented Treg induction. We also observed a reduced number of cDC1s and reduced PD-L1 expression in the lungs following secondary infection in the absence of CLEC-1, and these cells have been shown to be important for generating Tregs through TGFβ or PD-L1.^42^^,^^43^ We also found a reduced accumulation of PMNs following secondary infection in the absence of CLEC-1. These PMNs could correspond to myeloid-derived suppressor cells (MDSCs) harboring immunosuppressive properties and arising under these pathological conditions.^34^ Expansion of Tregs and MDSCs has been shown to contribute to immunoparalysis in sepsis.^5^ Tregs are known to suppress not only effector T cells, but also monocytes and neutrophil functionality during sepsis.^44^ Their blockade was reported to improve immunity and mortality rates in animal models of sepsis.^45^ MDSCs have been linked to the increased prevalence of nosocomial infections in patients with sepsis.^7^

Similarly, other inhibitory CLRs were described to be involved in the regulation of IFN response in DCs to finely polarize subsequent T cell activation during infections. The ITIM receptor CLEC12A, which binds monosodium urate crystals released by dying cells, inhibits inflammatory response following tissue damage to prevent immunopathology.^46^ In contrast, in the context of viral infection, CLEC12A has been reported to positively regulate the type I IFN response in DCs.^47^ Similarly, the ITIM receptor DCIR, which is particularly expressed by cDC2s, was shown to sustain type I IFN signaling in DCs, thereby inhibiting IL-12 production and Th1 cell differentiation during *Mycobacterium tuberculosis* infection.^48^ Furthermore, during *C. albicans* infection, DECTIN-1 has been reported to contribute to type I IFN expression by DCs, resulting in the recruitment of inflammatory cells, causing hyper-inflammation and lethal kidney pathology.^49^^,^^50^ In addition, during fungal infection, DECTIN-1 was shown in human DCs to lead to transient and intermediate expression of IFNβ, promoting the release of active TGFβ, which in turn resulted in the development of primarily non-pathogenic IL-17-expressing CD4^+^ T cells.^51^

Therefore, by sensing tissue integrity and by positively regulating the type I IFN response in cDC1s, CLEC-1 may provide a mechanism of cross-talk between PAMP- and DAMP-triggered signaling pathways at the early onset of sepsis to finely modulate the equilibrium between infection-driven inflammation and control of pathogens. CLEC-1 does not contain a classical ITAM or ITIM motif in the cytoplasmic tail but rather a tyrosine residue in a non-characterized signaling sequence [YSST] which could potentially be utilized for downstream signaling.^17^ We observed that the positive regulation of IFN response required CLEC-1 triggering by its ligands on necrotic cells and not to the infectious agent. Nevertheless, we observed that the absence of CLEC-1 signaling reduces the immunosuppressive state of these cells following infection, suggesting also a role of CLEC-1 in the regulation of immunosuppressive pathways following PAMP triggering. CLEC-1 is highly expressed in the lungs and was also shown to recognize DHN-melanin, a non-carbohydrate cell wall component found in many fungi, and to be essential for resistance to systemic infection by controlling fungal burdens in the lungs.^52^ To date, no study has reported CLEC-1 binding to glycan moieties expressed by commensal and pathogenic bacteria such as *E coli*. However, CLEC-1, following its triggering by tissue damage, could play a role by modulating the signaling pathways from other PRRs triggered by the bacteria to ensure microbial control while preserving the integrity of infected organs.

From a translational perspective, we demonstrated that blocking CLEC-1 signaling using an anti-human CLEC-1 mAb in CLEC-1 humanized mice mirrored the reduction of the excessive inflammation observed in *Clec1a* KO mice, except that these differences were noticed at the end of the acute phase of sepsis and concerned not only CXCL10 but also TNFα, CXCL1, and IL-1β levels. This suggests that the effect of CLEC-1 signaling blockade by the mAb may be delayed compared to the one observed by genetic ablation of *Clec1a*, explaining why no difference in body weight loss was observed at the early stage of sepsis by mAb blockade. This may be due to a partial or different CLEC-1 signaling blockade with the mAb. This could also be attributed to different kinetics of sepsis between the genetic background of CLEC-1 humanized mice and that of *Clec1a* KO mice.

To conclude, our results demonstrate that the innate receptor CLEC-1 promotes the early IFN response in lung cDC1s, potentiates monocyte infiltration, and establishes immunosuppression in a mouse model of sepsis. Moreover, we highlight the therapeutic potential of human CLEC-1 targeting with a mAb to prevent sepsis-induced dysregulation and identified *CLEC1A-expressing* cDC1s in lung from patients to support the clinical relevance of CLEC-1 targeting following infection.

### Limitations of the study

In this study, we used mice with a global *Clec1a* deficiency, and although we observed that the primary effect on the phenotype was on cDC1s—the myeloid cell subtype that expresses the highest levels of *Clec1a* during sepsis— we cannot completely rule out indirect contributions of CLEC-1 in other myeloid cell subtypes or in endothelial cells. A conditional deletion of *Clec1a* in cDC1s will be necessary to definitively attribute the phenotype observed *in vivo* to the intrinsic function of CLEC-1 in cDC1s. Furthermore, *in vitro* assessment of the response of purified cDC1s to necrotic cells or IFN may not replicate the integration of multiple external signals occurring *in vivo* during sepsis and thus may not precisely reveal the intrinsic role of CLEC-1 in cDC1s. Another limitation of this study is that a single dose and a single injection were used to block CLEC-1 with the mAb, which may explain some of the discrepancies observed in comparison with *Clec1a* KO mice. Different doses and serial injections could be evaluated to optimize the effect on inflammatory response during sepsis induced by *E. coli* but also by Gram-positive bacteria or by polymicrobial infections. Moreover, although this mAb could represent a therapeutic tool, it remains to establish the expression and function of CLEC-1 in human bacterial sepsis. Despite these limitations, our findings provide valuable insights into CLEC-1-mediated regulation of the inflammatory response in the context of sepsis.

## Resource availability

### Lead contact

Requests for further information and resources should be directed to and will be fulfilled by the lead contact, Elise Chiffoleau (elise.chiffoleau@univ-nantes.fr).

### Materials availability

Anti-human CLEC-1 mAbs and human CLEC-1 KI mice could be provided by Nantes Université and OSE Immunotherapeutics pending scientific review (depending on the research subject, university, potential of valorization, and so forth) and a completed material transfer agreement. Requests should be submitted to: elise.chiffoleau@univ-nantes.fr and aurore.morello@ose-immuno.com.

### Data and code availability

• ScRNA-seq and bulk RNA-seq data have been deposited at GEO: GSE326200 and GSE326909, respectively, and are publicly available.
• Data analysis code for scRNA-seq and bulk RNA-seq data is available at Zenodo https://doi.org/10.5281/zenodo.17737536 and https://doi.org/10.5281/zenodo.17750854 respectively and are publicly available.
• Any additional information required to reanalyze the data reported in this paper is available from the lead contact upon request.

## Acknowledgments

We acknowledge the CIPHE unit (INSERM, CNRS, Aix-Marseille University, France) for the generation of the hCLEC-1 KI mouse. This work was supported by ANR generique PRC 2022 (ANR-22-CE17-0053, CLIPS) from National Research Agency (ANR) for French government.

## Author contributions

Conceptualization, A.M., R.J., A.M., E.F., N.P., J.P., A.R., C.J., and E.C.; investigation, T.T.O., E.P., J.S., M.D., V.G., C.L., F.P.M., C.F., C.C., J.M., and M.B.; supervision A.R., C.J., and E.C.; writing – original draft, T.T.O. and E.C.; review and editing, E.C.

## Declaration of interests

E.C. and N.P. are authors of patents related to CLEC-1 inhibition. N.P., A. M., and E. F. are employees and shareholders of OSE Immunotherapeutics, a company involved in immunotherapy development. The other authors declare no competing interests.

## STAR★Methods

### Key resources table

**Table:** 

REAGENT or RESOURCE	SOURCE	IDENTIFIER
**Antibodies**		

CD11b BV421 (clone M1/70),	BD Biosciences	Cat#562605; RRID: AB_11152949
CD11b BV605 (clone M1/70)	Biolegend	Cat#101237; RRID: AB_2565431
CD11c PE-Cy7 (clone N418)	Biolegend	Cat#117317; RRID: AB_493568
CD16/CD32 (Fc Block) Unconjugated (clone 2.4G2)	BD Biosciences	Cat#553142; RRID: AB_394657
CD24 BV711 (clone M1/69)	BD Biosciences	Cat#563450; RRID: AB_2738213
CD274 BV711 (clone MIH5)	BD Biosciences	Cat#563369; RRID: AB_2738163
CD3e BV711 (clone 145-2C11)	BD Biosciences	Cat#563123; RRID: AB_2687954
CD4 BUV395 (clone GK1.5)	BD Biosciences	Cat#563790; RRID: AB_2738426
CD40 BV605 (clone 3/23)	BD Biosciences	Cat#745218; RRID: AB_2742809
CD44 BV480 (clone IM7)	BD Biosciences	Cat#566200; RRID: AB_2739518
CD45 BV786 (clone 30-F11)	BD Biosciences	Cat#564225; RRID: AB_2716861
CD45 R718 (clone 30-F11)	BD Biosciences	Cat#567075; RRID: AB_2916421
CD45 BV480 (clone 30-F11)	BD Biosciences	Cat#566095; RRID: AB_2739499
CD45 FITC (clone 30-F11)	BD Biosciences	Cat#553079; RRID: AB_394609
CD74 BV786 (clone In-1)	BD Biosciences	Cat#740939; RRID: AB_2740569
CD8 APC (clone 53–6.7)	BD Biosciences	Cat#553035; RRID: AB_398527
CD80 PE (clone 16-10A1)	BD Biosciences	Cat#553769; RRID: AB_395039
CD86 FITC (clone GL1)	BD Biosciences	Cat#553691; RRID: AB_394993
CLEC9A BUV395 (clone 7H11)	BD Biosciences	Cat#752673; RRID: AB_2917656
CXCR6 PE-D594 (clone SA051D1)	Biolegend	Cat#151116; RRID: AB_2721700
F4/80 PE-Cy5.5 (clone BM8)	eBioscience	Cat#15-4801-82; RRID: AB_468798
F4/80 A647 (clone BM8)	Biolegend	Cat#123121; RRID: AB_893480
FoxP3 FITC (clone FJK-16s)	eBioscience	Cat#11-5773-82; RRID: AB_465243
I-A/I-E BUV737 (clone M5/114)	BD Biosciences	Cat#748845; RRID: AB_2873248
IFNAR1 PE (clone MAR1-5A3)	Biolegend	Cat#127312; RRID: AB_2248800
IgG1 κ Iso PE (clone MOPC-21)	BD Biosciences	Cat#555749; RRID: AB_396091
Ly6C R718 (clone AL-21)	BD Biosciences	Cat#566987; RRID: AB_2869991
Ly6C BV421 (clone AL-21)	BD Biosciences	Cat#562727; RRID: AB_2737748
Ly6G R718 (clone 1A8)	BD Biosciences	Cat#567039; RRID: AB_2916402
Ly6G A647 (clone 1A8)	Biolegend	Cat#127610; RRID: AB_1134159
NK1.1 BV421 (clone PK136)	BD Biosciences	Cat#562921; RRID: AB_2728688
SiglecF PE (clone S17007L)	Biolegend	Cat#155505; RRID: AB_2750234
γδTCR PE-Cy7 (clone GL3)	Biolegend	Cat#118123; RRID: AB_11204423
TNFα PE (clone MP6-XT22)	BD Biosciences	Cat#554419; RRID: AB_395380
XCR1 APC (clone ZET)	Biolegend	Cat#148206; RRID: AB_2563932
Anti-human CLEC-1 antagonist IgG4 mAbs	OseImmunotherapeutics	N/A
Isotype control IgG4 mAb (clone b12)	OseImmunotherapeutics	N/A

**Bacterial and virus strains**		

E. coli (strain DH5α)	ATCC	53868
YFP-E. coli with p-HG-1 plasmid)	In house (from ATCC strain DH5α)	N/A

**Chemicals, peptides, and recombinant proteins**		

Collagenase IV	Roche	Cat# 11088882001
Dispase	Corning	Cat# 354235
recombinant mouse IFNβ	BioTechne R&D Systems	Cat# 8234-MB-010
TLR3-L (poly(I:C))	InvivoGen	Cat# tlrl-picw
TLR4-L (LPS)	Sigma-Aldrich	Cat# L2630
Collagenase III	Stem Cell technologies	Cat# 07422
DNase I	Sigma Aldrich	Cat# DN25
e780 viability dye	eBioscience	Cat# 65-0865-14
Precision counting beads	BioLegend	Cat# 424902
Roswell Park Memorial Institute medium (RPMI) 1640 medium	Gibco	Cat# 11875093
10% endotoxin-free fetal bovine serum	Thermo Fisher Scientific	Cat# A5209501
L-glutamine	Sigma-Aldrich	Cat# G8540
Pénicilline-streptomycine (10 000 U/ml)	Gibco	Cat# 11548876

**Critical commercial assays**		

LEGENDplex™ Mouse Anti-Virus Response Panel (13-plex)	Biolegend	Cat# 740621
DC isolation kit	Miltenyi	Cat# 130-091-169
BD GolgiPlug	BD Biosciences	Cat# 555029
BD CytoFix Fixation Buffer	BD Biosciences	Cat# 554655
Chromium Single Cell 3′ Library & Gel Bead Kit v2	10X Genomics	Cat# PN-120267
Chromium Next GEM Single Cell 3′ GEM, Library & Gel Bead Kit v3.1	10X Genomics	Cat# PN-1000121

**Deposited data**		

scRNA-seq data	generated in this study	Accession GEO: GSE326200
bulk RNAseq data	generated in this study	Accession GEO: GSE326909
data analysis code of scRNA-seq data	generated in this study	Accession: Zenodo https://doi.org/10.5281/zenodo.17737536
data analysis code of bulk RNAseq data	generated in this study	Accession: Zenodo https://doi.org/10.5281/zenodo.17750854

**Experimental models: Cell lines**		

MCA101-FcROVA C57Bl/6 fibrosarcoma cells expressing mOVA (MCA-mOVA)	C. Thery, Curie, Paris, France	N/A

**Experimental models: Organisms/strains**		

C57BL/6NCrl-Clec1a^tm1(EGFP,−cre/ERT2)Djr^ knock-in (KI)/knock-out mice	D. J. Rossi, Harvard, USA	N/A
C57BL/6NRjClec1a^tm1Ciphe^ mice	E Chiffoleau, Nantes, France	N/A

**Software and algorithms**		

Cellranger	10X Genomics	7.2.0
Seurat package ().	Seurat	v4.4.0
fgsea package ()	fgsea	v.1.32.4
ggplot2 package	ggplot2	v3.5.2
Qognit	LegendPlex	web application
FlowJo.	Windows, Ashland	v10.10
BioRender	graphical illustrations	web tool Graphical abstract: Created in BioRender. Chiffoleau, E. (2026) https://BioRender.com/hz4jh0l Figure 1A: Created in BioRender. Josien, R. (2026) https://BioRender.com/oe48ln8. Figure 2A: Created in Biorender. Josien, R. (2026) https://BioRender.com/oe48ln8. Figure 3A: Created in BioRender. Josien, R. (2026) https://BioRender.com/oe48ln8. Figure 4A: Created in BioRender. Josien, R. (2026) https://BioRender.com/oe48ln8. Figure 5B was created in BioRender. Josien, R. (2026) https://BioRender.com/oe48ln8.
GraphPad Prism v11.0.1	GraphPad software	N/A

**Other**		

10X Chromium Controller	10× Genomics	N/A
NovaSeq 6000	Illumina	SFR Bonamy
BD Fortessa X-20 cytometer.	BD Biosciences	SFR Bonamy

### Experimental model and study participant details

For the study, we used C57BL/6NCrl-Clec1a^tm1(EGFP,−cre/ERT2)Djr^ knockin (KI)/knockout (KO) mice rederived from sperm (kindly provided by D. J. Rossi, Harvard, USA) and CLEC-1 humanized KI mice (C57BL/6NRjClec1a^tm1Ciphe^) generated by “Center d’immuno-phénomique” (Marseille, France). Mice were used at 8 to 12 weeks old (20–25 g), and littermates were used for experiments. Mice were used according to experimental procedures carried out in strict accordance with protocols approved by the Committee on the Ethics of Animal Experiments of Pays de la Loire and authorized by the French Government’s Ministry of Higher Education and Research (Apafis no. 27741R 28 01 2021). For clinical parameters and flow cytometry studies, both males and females were used. For scRNAseq and RNAseq experiments, only males were used for reproducibility of the experiments. For infection, we used a strain of *E. coli* DH5-α (ATCC 53868) routinely used in the laboratory. MCA101-FcROVA C57Bl/6 fibrosarcoma cell line expressing mOVA (MCA-mOVA) (kindly provided by C. Thery, Curie, Paris, France) was routinely tested for mycoplasma contamination by Polymerase Chain Reaction.

### Method details

#### Animals and genotyping

C57BL/6NCrl-Clec1a^tm1(EGFP,−cre/ERT2)Djr^ knockin (KI)/knockout (KO) mice (referred as *Clec1a* KO as homozygotes) (by insertion of *eGFP-CreERT2* into *Clec1a* to generate null alleles) were rederived from sperm (kindly provided by D. J. Rossi, Harvard, USA) (Gazit et al., 2014) and littermates WT mice were used as controls. CLEC-1 humanized KI mice (C57BL/6NRjClec1a^tm1Ciphe^) were generated by “Center d’immuno-phénomique” (Marseille, France) by humanization of the mouse *Clec1a* molecule. Briefly, a hybrid CLEC1A cDNA (human extracellular domain of *CLEC1A* appended to mouse sequences coding for the transmembrane and cytosolic domains of *Clec1a*) was introduced in the first coding exon of *Clec1a* mouse locus (Clec1a-201 ENSMUST00000037481.9 transcript). PCR genotyping of *Clec1a* KO and hCLEC-1 KI mice was performed according to protocols previously described (Drouin et al., 2022). At steady state, *Clec1a* KO and hCLEC-1 KI mice exhibit no difference in immune cell composition compared to regular naive mice (Drouin et al., 2022). Mice were bred in Center de Distribution, Typage et Archivage animal (Orléans, France) under specific pathogen–free conditions and used at 8 to 12 weeks old (20–25 g), and littermates were used for experiments. For animal studies, experimental procedures were carried out in strict accordance with protocols approved by the Committee on the Ethics of Animal Experiments of Pays de la Loire and authorized by the French Government’s Ministry of Higher Education and Research (Apafis no. 27741R 28 01 2021). Animals were anesthetized with a mixture of 5% isoflurane and oxygen at 1 L/min and euthanized by pentobarbital injection or cervical dislocation after anesthesia.

#### *E. coli* infection and treatment

One day before infection, a strain of *E. coli* DH5-α (ATCC 53868) was cultured overnight in Brain-Heart Infusion (BHI) broth at 37 °C under slow agitation and the concentration was measured with a McFarland densitometer, and adjusted to obtain a suspension at an optic density of 7 McF (10^9^ Colony Forming Units (CFU)/mL). Then, C57BL/6 *Clec1a* KO or WT mice, or humanized CLEC-1 KI mice were intratracheally injected under anesthesia with 75 μL of this *E. coli* DH5-α suspension and monitored every day from day 1–7 for primary infection and from day 7 + 1 to 7 + 7 for secondary infection for their body weight. Humanized CLEC1 mice were treated with a humanized anti-human CLEC-1 antagonist IgG4 mAbs (generated by OseImmunotherapeutics, France) or with isotype control IgG4 mAb (OseImmunotherapeutics, France) (100 μg per mouse) intraperitoneally 5 min prior to primary infection.

#### Bacterial burden assay

C57BL/6 *Clec1a* WT or KO mice were infected with 75 μL of an *E. coli* DH5-α suspension (7 McF) as described. Two days after infection, lungs were removed, weighed, and homogenized in 1 mL of saline buffer (Mixer Mill MM 400, Retsch, Newtown, PA) and used for quantitative cultures on agar (BD BBL CHROMagar Orientation and BD Trypticase Soy Agar II) for 24 h at 37°C. Viable counts, measured 48 h after incubation, were expressed as mean (±SD) log_10_ CFU per gram of organ.

#### Lung dissociation protocol

Mice were euthanized by pentobarbital lethal injection at day 1, 3 or 7 post-primary or secondary infection and blood was collected by intracardiac puncture. For lung dissociation, blood was first purged by injecting PBS through the right ventricle of the heart. Then, lungs were instilled intratracheally with 3 mL of dispase solution (Corning, cat: 354235) at a 1:10 dilution in HBSS, followed by agarose to enhance enzyme penetration in the tissue. Lungs were then recovered and placed into a tube containing 3 mL of dispase diluted by 1:10 in HBSS (Gibco), for 30 min at room temperature. After mechanical dissociation, the cell suspension was filtered through 100 μm and 40 μm cell strainers.

#### *In vivo* phagocytosis assay

YFP-*E. coli* (strain DH5α with p-HG-1 plasmid) were grown overnight in BHI broth media with 20 μg mL^−1^ chloramphenicol. Fluoresbrite YG carboxylate microspheres (3.64 × 10^9^ beads, 0.5 mm; Polysciences) or YFP-*E. coli* (OD_600_ = 2–3, 75 μL) were injected intratracheally in mice. After 2 h, the percentages of YFP^+^ macrophages in the lungs were measured by flow cytometry.

#### cDC1 purification and stimulation

Spleen from WT and *Clec1a* KO mice were recovered, minced and incubated with collagenase IV for 45 min at 37°C (Roche, cat: 11088882001). The resulting cell suspension was then enriched in cDC1s by incubation with a DC isolation kit (Miltenyi, cat: 130-091-169) and sorted with an AutoMACS separator. The resulting cell suspension was then stained with fluorochrome-conjugated antibodies and purified cDC1s were sorted by FACS (based on the expression of CD11c, MHC-II and XCR1) using a Cytek Aurora spectral flow cytometer. Purified cDC1s were then plated in flat-bottom 96-well plates in Roswell Park Memorial Institute medium (RPMI) 1640 medium (Gibco), supplemented with 10% endotoxin-free fetal bovine serum (Thermo Fisher Scientific), 2 mM L-glutamine (Sigma-Aldrich, cat: G8540), 100 U/ml penicillin and 100 μg/mL streptamycin (GIBCO, cat: 11548876) at 37°C and 5% CO2. Then, cDC1s were stimulated 6h (for RNAseq experiments) or 17h (for phenotype, cytokine and chemokine expression) with *E. coli* (MOI = 10), necrotic cells (MCA101-FcROVA C57Bl/6 fibrosarcoma cells expressing mOVA (MCA-mOVA) (kindly provided by C. Thery, Curie, Paris, France) treated 18 h earlier by UV-C light (300 mJ/cm2; CL-1000 UV Crosslinker, Analytik Jena)) at ratio 1:10) alone or together with TLR3 L (20 μg/mL, poly:((I:C) LMW, InvivoGen cat tlrl-picw), TLR4 L (0.1 μg/mL, LPS, Sigma-Aldrich, cat:L2630) or recombinant mouse IFNβ (1000 U/ml, BioTechne R&D Systems cat: 8234-MB-010). Then, cells were extensively washed, harvested and analyzed by flow cytometry or RNA sequencing (RNAseq).

#### Assessment of cytokines and chemokines

Blood from WT and *Clec1a* KO mice were recovered by intracardiac puncture immediately after euthanasia by pentobarbital injection. Blood samples were allowed to clog at room temperature for 50 min and sera were collected after centrifugation at 11,000g for 10 min at room temperatures. Supernatants from purified cDC1s stimulated *in vitro* were harvested 17h following stimulation. The level of cytokines and chemokines from the sera and supernatants were assessed using the LEGENDplex Mouse Anti-Virus Response Panel (13-plex) (BioLegend cat# 740621) and quantified by flow cytometry. Data were analyzed via the Qognit LegendPlex web application.

#### *In vivo* and *ex vivo* stimulation with recombinant IFNβ

For *in vivo* stimulation, WT and *Clec1a* KO mice were injected intravenously via the caudal vein with 30,000 UI of mouse recombinant IFNβ (Biotechne R&D Systems cat: 8234-MB-010). After 17h, the spleen of the mice was recovered following mouse euthanasia by pentobarbital lethal injection. Spleen was then minced and incubated for 45 min at 37°C in a collagenase III (StemCell technologies cat: 07422) and DNAse I (Sigma-Aldrich cat: DN-25). Then, the tissue was filtered through a 70 μm cell strainer obtaining a cell suspension for flow cytometry. For *ex vivo* stimulation, cells dissociated from the spleen of WT and *Clec1a* KO mice, were plated in 24-well plates and stimulated with 3,000 U/mL of mouse recombinant IFNβ (Biotechne R&D Systems cat: 8234-MB-010) for 17h.

#### Flow cytometry analysis

For extracellular staining, cells were first incubated with CD16/CD32 Fc Block, then with fluorochrome-conjugated antibodies and e780 viability dye (eBioscience, cat 65-0865-14) for 30 min at 4°C. For intracellular staining, cells were first stimulated with PMA/ionomycin in RPMI culture medium for 1 h at 37°C, followed by incubation with BD GolgiPlug (BD Biosciences cat: 555029) (diluted 1:1000 in RPMI) for 3 h to block exocytosis. Cells were then permeabilized with BD CytoFix Fixation Buffer (BD Biosciences cat n°554655) for 1h at room temperature and stained with flurochrome-conjugated antibodies for 30 min at 4°C. To determine the cell count per organ, precision counting beads (BioLegend cat: 424902) were added to each well prior to flow cytometric acquisition. Data were acquired with a BD Fortessa X-20 cytometer (BD Biosciences). Flow cytometry data was analyzed with FlowJo (v10.10, Windows, Ashland) and expressed in geometric Mean Fluorescence Intensity (MFI).

#### Single-cell RNA sequencing (scRNAseq) sample preparation

The lungs of uninfected (*n* = 2) and infected mice at day 1 (*n* = 2), 3 (*n* = 4) or 7 (*n* = 2) following primary or secondary infection were dissociated as described above. The obtained cell suspension was then incubated 15 min at 4°C with CD16/CD32 Fc Block, and then 30 min at 4°C with e780 viability dye, anti-CD45/FITC, antiLy6G/Alexa 647, and anti-CD31/BV421 to FACS-sort CD45^+^, CD45^+^Ly6G^−^ and CD45^−^CD31^+^ cells (BD FACS ARIA, BD Biosciences). Then, in order to reduce the predominant neutrophil population and to enrich in endothelial cells for scRNAseq experiments, purified cells were mixed with as follow (6·10^5^ CD45^+^ Ly6G^−^ cells, 3·10^5^ CD45^+^ cells and 1·10^5^ CD45^−^ CD31^+^ cells).

#### ScRNAseq library preparation, sequencing and data analysis

Cells were incubated with TotalSeq barcoded-antibodies for each sample, in order to identify their origin after RNA library creation. Approximately 16,000 cells per reaction were processed using a 10X Chromium Next Controller following the 3′ V3 protocol. RNA libraries were then prepared using the Chromium Single Cell 3′ Library and Gel Bead Kit v2 (10X Genomics, cat: PN-120267) and Chromium Next GEM Single Cell 3′ Library and Gel Bead Kit v3.1 (10X Genomics, cat: PN-1000121). Libraries were then sequenced with an Illumina NovaSeq 6000 (SFR Bonamy). Sequenced data were processed using the Cellranger-7.2.0 pipeline for sample demultiplexing, sequence alignment, barcode processing and unique molecular identity (UMI) counting. Filtered matrixes were processed and cells containing more than 5% of mitochondrial genes were filtered off. Dataset integration, clustering and differential gene expression was performed using the Seurat package (v4.4.0). Gene Set Enrichment Analysis (GSEA) was performed using the fgsea package (v.1.32.4). GSEA results were visualized using ggplot2 (v3.5.2).

#### Mini-bulk RNAseq library preparation, sequencing and data analysis

Treated cDC1s were resuspended in 4 technical replicates per condition (*n* = 6 mice per genotype) at a concentration of 1,250 cells/uL in lysis buffer containing 0.16μL 10% Triton X-100, 0.08 μL RNAse Out (40U/μL, Invitrogen) and 3.75 μL RNAse DNAse free water. A total of 5,000 cells per well (=4 μL cell suspension/well) were then immediately loaded on a 96-plate using an OT-II library prep robot (Opentrons). Plates were then stored at −80°C for a few days. 1uL barcoded RT-primers with universal adapters, well-specific barcodes, and unique molecular identifiers were then added to the wells and incubated at 72°C for 3 min before reverse transcription. As previously described (Chaumette et al., 2022; Letellier et al., 2022), this method allows the mRNA poly(A) tails to be tagged during template-switching reverse transcription. Barcoded complementary DNAs from multiple samples were then pooled, amplified, and tagmented using a transposon fragmentation approach which enriches for 3′ ends of complementary DNA. A library of 350–800 bp was run on a 100-cycle 1.5B flow cell on the NOVAseq X-plus series at the Genomics Antlantic platform (Nantes) facility (Nantes). An average of 5 million 75 bp single-end reads were obtained for each sample. Samples were demultiplexed and aligned to the GRCm38 (mm10) genome using the DGE bioinformatics pipeline. Post-sequencing, data demultiplexing was performed using Illumina bcl2fastq. The resulting FASTQ files were analyzed using the 3′ Sequencing RNA Profiling (SRP) pipeline (Charpentier et al., 2021). This methodology aligns with previous DGEseq analysis in various studies (Chaumette et al., 2022; Letellier et al., 2022). Differential gene expression (DGE) analysis was performed by using the DESeq2 package (v1.46.0). Gene Set Enrichment Analysis (GSEA) was performed by using the fGSEA package (v.1.32.4). Visualisation of DEG volcano plots and GSEA results were obtained by using the ggplot2 package (v3.5.2).

### Quantification and statistical analysis

Apart experiments for RNA sequencing, the sample size calculation for *in vivo* and *in vitro* experiments was performed with the online software (Experimental design assistant https://eda.nc3rs.org.uk/eda/login/auth) according to the estimated effect size and standard deviation (Variability base from previous data) for the different non-parametric tests (Mann-Whitney) with *p* < 0.05 and power experiment set to 0.90. Except for *in vivo* mAb treatment, all results were obtained in at least 2 individual experiments and statistical analyses were performed using Graphpad Prism 10 (La Jolla, CA, USA) using the Mann-Whitney *t* test. *p* values <0.05 were considered significant and statistical details of experiments can be found in the figure legends.
