# Supplementary material for: CLEC-1 promotes early IFN response in cDC1s and favors immune dysregulation during sepsis in mice
Source: iScience. 2026 Aug 24;29(9):117239. doi: 10.1016/j.isci.2026.117239 (PMC13524758; doi:10.1016/j.isci.2026.117239)
Supplement: Supplementary file 1 [file mmc1.pdf]

iScience, Volume *29*

Supplemental information

CLEC-1 promotes early IFN response in cDC1s and favors immune dysregulation during sepsis in mice

Thomas Teja Ogor, Esther Porée, Javier Saenz, Marion Davieau, Victor Gourain, Camille Ligeron, Florian Pierre Martin, Cynthia Fourgeux, Claire Chédeville, Julie Mignon, Martin Braud, Aurélie Moreau, Régis Josien, Aurore Morello, Etienne Foucher, Nicolas Poirier, Jeremie Poschmann, Antoine Roquilly, Cédric Jacqueline, and Elise Chiffoleau

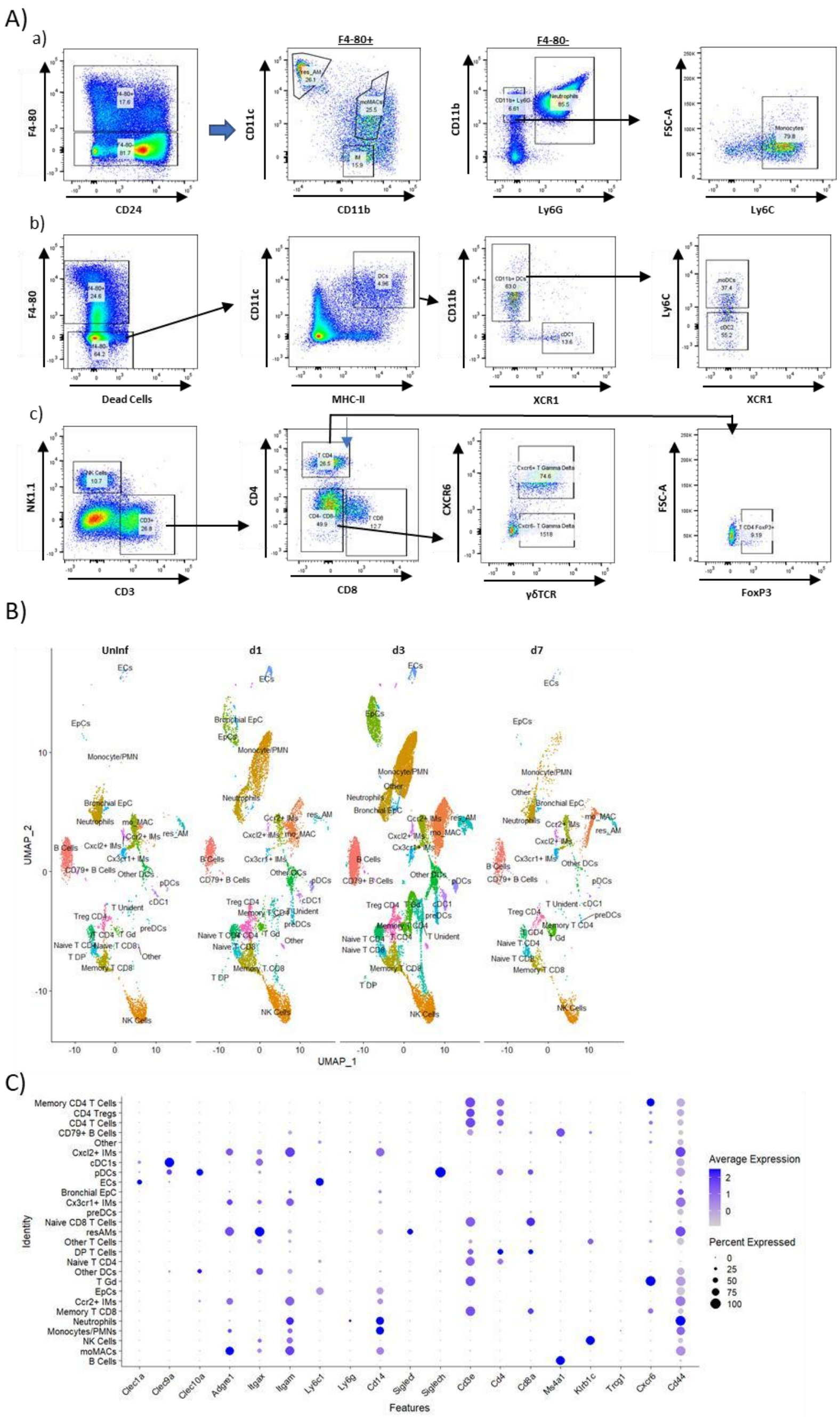

Figure S1: Identification of different immune cell subtype populations from the lung during sepsis in mice by flow cytometry and scRNAseq analysis.

A) Flow cytometry cell gating strategy represented by pseudocolor plots of relevant cell markers used for the identification of **a)** lung resident alveolar macrophages (resAMs) (CD11c-hi, CD11b-lo, F4-80+, SiglecF+), interstitial macrophages (IMs) (CD11c-, CD11b-med, F4-80+), monocyte-derived macrophages (moMACs) (CD11c-lo, CD11b-hi, F4-80+), polymorphonuclear (PMNs) (F4-80-, CD11b+, Ly6G+), and monocytes (F4-80-, CD11b+, Ly6G-, Ly6C+). **b)** cDC1s (F4-80-, CD11c-hi, MHC-II-hi, CD11b-, XCR1+), cDC2s (F4-80-, CD11c-hi, MHC-II-hi, CD11b+, XCR1-, Ly6C-) and moDCs (F4-80-, CD11c-hi, MHC-II-hi, CD11b+, XCR1-, Ly6C+), **c)** NK cells (CD3-, NK1.1+), CD4 T (CD3+, CD4+), CD8 T (CD3+, CD8+), γδ T (CD3+, γδTCR+) and Tregs (CD3+, CD4+, FoxP3+). **B)** Visualization of a Uniform Manifold Approximation and Projection (UMAP) plot obtained with the Seurat R package illustrating the different scRNAseq clusters annotated from mouse lungs following intratracheal *E. coli* infection in uninfected (uninf) condition and at day 1, 3 and 7 post-primary infection. Cells derived from n=2 mice for uninf, d1 and 7 and from n=4 mice for d3 for each genotype. **C)** Dotplot representation of several canonical markers used for clustering identification for scRNAseq analysis.

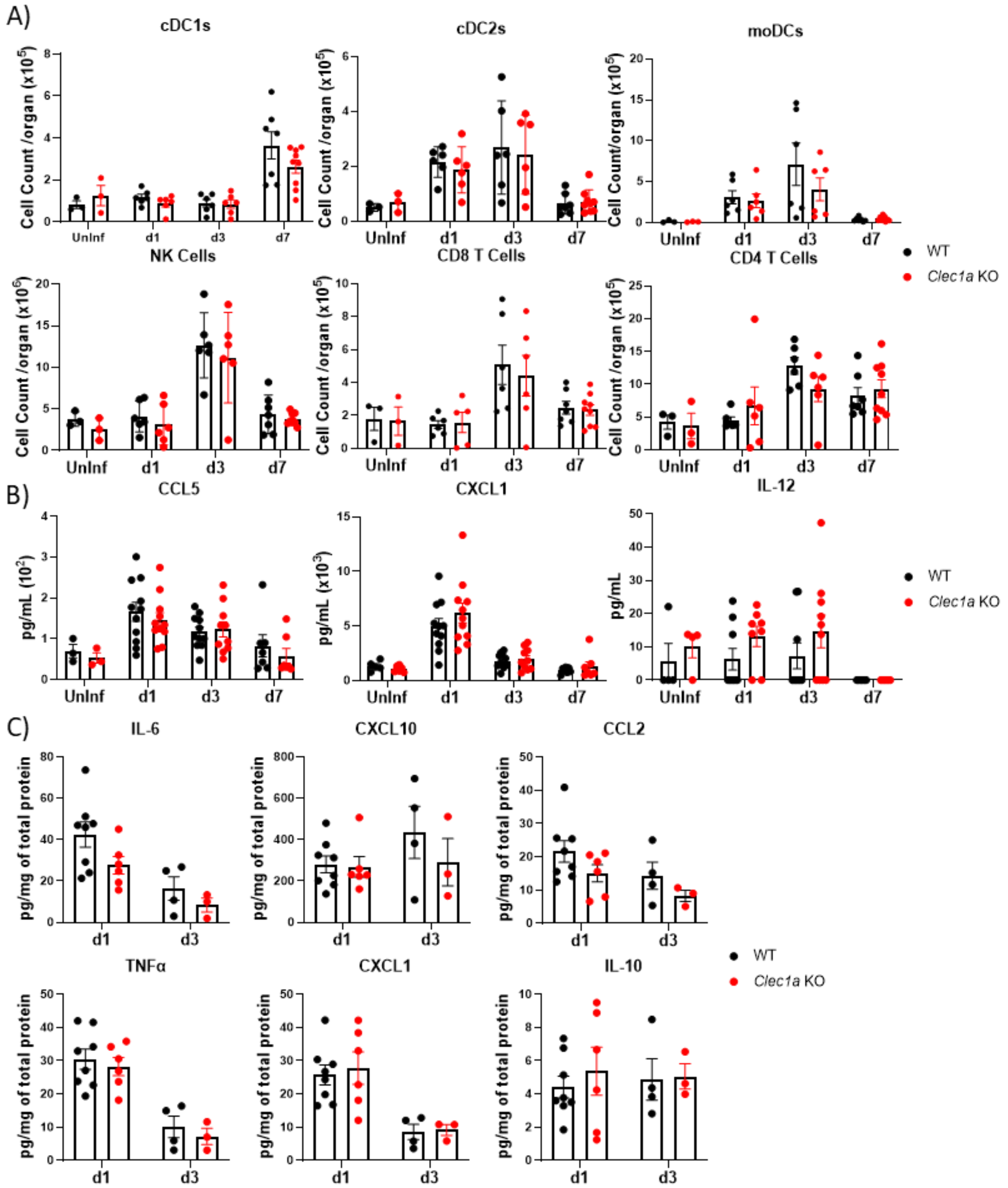

Figure S2: Cell count per lung of immune cell populations and concentration of cytokines and chemokines in serum and lungs following E. coli infection in WT and *Clec1a* KO mice.

A) Flow cytometry cell count per lung of monocyte derived dendritic cells (moDCs), type II conventional dendritic cells (cDC2), natural killer cells (NK), CD8 and CD4 T cells from WT and *Clec1a* KO mice in uninfected condition (Uninf) or at day 1, 3 and 7 after intratracheal *E. coli* infection (Uninf n=3 for each genotype; d1-d7 n=6 for each genotype from two independent experiments) and presented as mean ± SEM. **B)** Cytokines and chemokines were analyzed by flow cytometry using a multiplex bead-based assay from the sera of WT and *Clec1a* KO mice in uninfected condition (Uninf) and at day 1, 3 and 7 after intratracheal *E. coli* infection (Uninf n=3-4 for each genotype; d1-d7 n=11 for each genotype from two independent experiments) and presented as mean ± SEM. **C)** Cytokines and chemokines were analyzed by flow cytometry using a multiplex bead-based assay from the lungs of WT and *Clec1a* KO mice at day 1 and 3 after intratracheal *E. coli* infection and data were normalized by total amount of lung protein and presented as mean ± SEM (n=8 for WT and n=6 for *Clec1a* KO at d1; n=4 for WT and n=3 for *Clec1a* KO at d3 from two independent experiments).

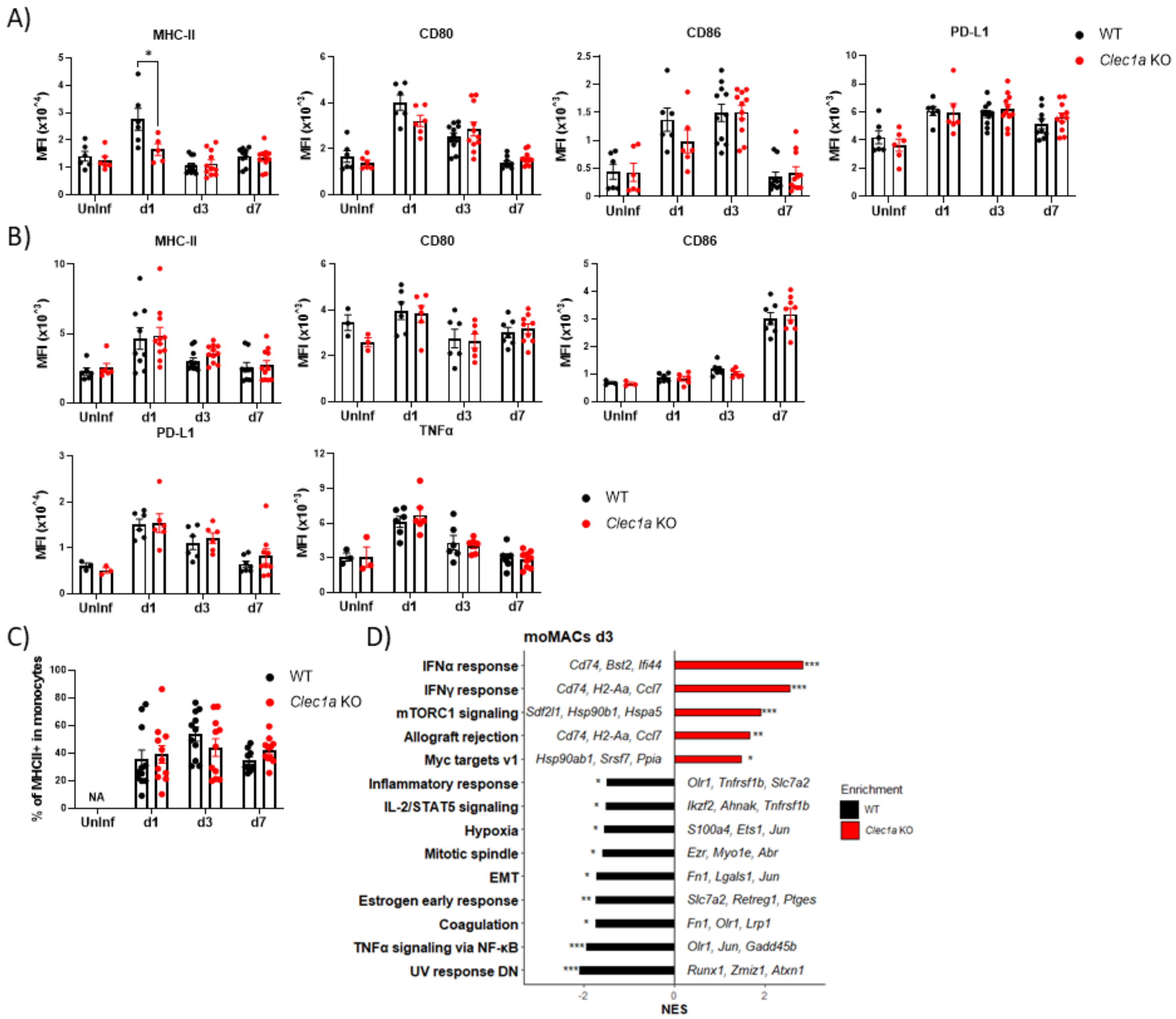

Figure S3: Characterization of lung immune cell populations following *E. coli* infection in WT and *Clec1a* KO mice.

A) Expression of activation markers on lung cDC2s, **B)** on lung resident alveolar macrophages (resAMs) and **C)** percentage of MHC-II positive cells in monocytes in the lungs from WT and *Clec1a* KO mice in uninfected condition (Uninf) and at day 1, 3 and 7 following intratracheal *E. coli* infection (n=6 for Uninf and at d1 for each genotype; n=8-11 for d3 and 7 for each genotype from two independent experiments). Data were assessed by flow cytometry and represented by the Median Fluorescence Intensity (MFI) or in percentage as mean ± SEM, *p<0.05 by Mann-Whitney t-test. **D)** Gene set enrichment analysis (GSEA) showing the Hallmark Pathways significantly enriched in the scRNAseq cluster of lung moMACs from WT and *Clec1a* KO mice at day 3 following intratracheal *E. coli* infection (n=4 mice). GSEA results represent the Normalized Enrichment Score (NES) and the adjusted p-value was estimated by an adaptive multi-level split Monte-Carlo scheme used by the fgsea package, *p<0.05, *p<0.01, ***p<0.001.

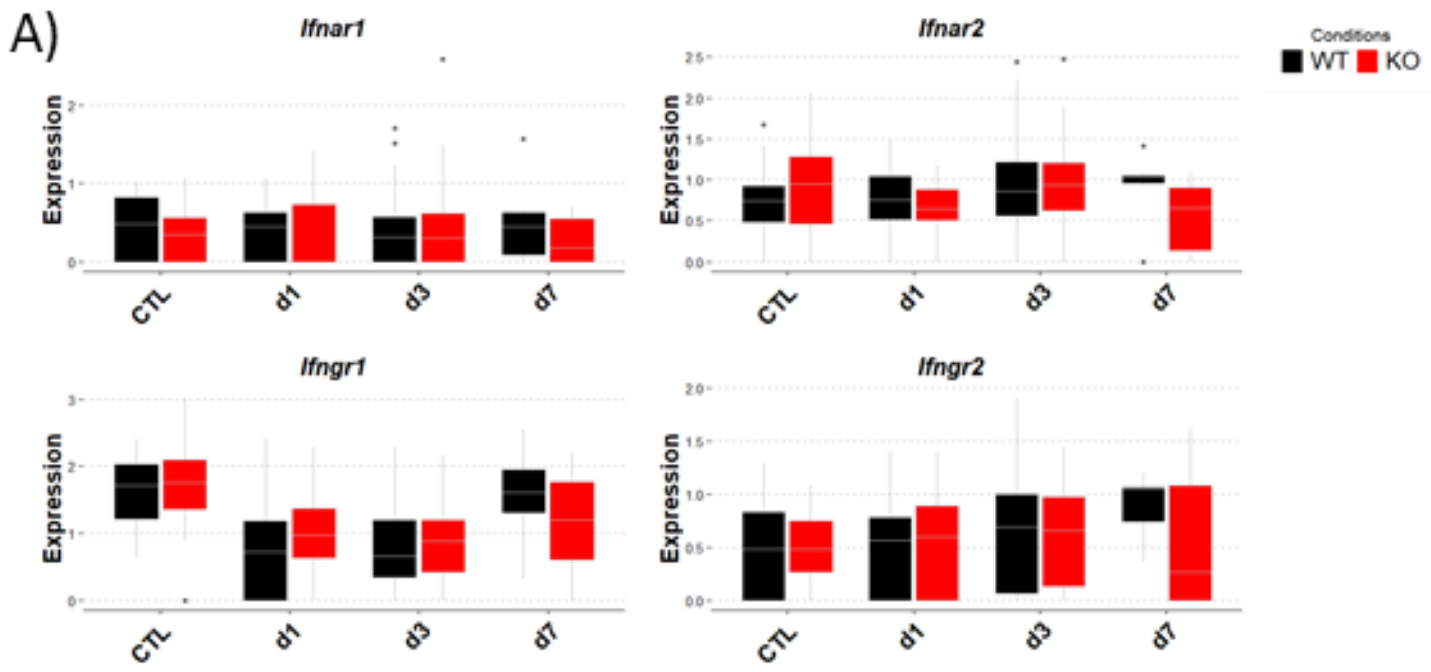

Figure S4: IFN receptor gene expression in cDC1s from WT and *Clec1a* KO mice during sepsis. Box plot of scRNAseq analysis illustrating *Ifnar1*, *Ifnar2*, *Ifngr1* and *Ifngr2* gene expression in lung cDC1s from WT and *Clec1a* KO mice at control steady state (CTL) or at day 1, 3 and 7 following intratracheal *E. coli* infection.
